# Physiological Processes Modulated by the Chloride-Sensitive WNK-SPAK/OSR1 Kinase Signaling Pathway and the Cation-Coupled Chloride Cotransporters

**DOI:** 10.3389/fphys.2020.585907

**Published:** 2020-10-20

**Authors:** Adrián Rafael Murillo-de-Ozores, María Chávez-Canales, Paola de los Heros, Gerardo Gamba, María Castañeda-Bueno

**Affiliations:** ^1^Department of Nephrology and Mineral Metabolism, Instituto Nacional de Ciencias Médicas y Nutrición Salvador Zubirán, Mexico City, Mexico; ^2^Facultad de Medicina, Universidad Nacional Autónoma de México, Mexico City, Mexico; ^3^Unidad de Investigación UNAM-INC, Instituto Nacional de Cardiología Ignacio Chávez and Instituto de Investigaciones Biomédicas, Universidad Nacional Autónoma de México, Mexico City, Mexico; ^4^Unidad de Investigación UNAM-INC, Research Division, Facultad de Medicina, Universidad Nacional Autónoma de México, Mexico City, Mexico; ^5^Molecular Physiology Unit, Instituto de Investigaciones Biomédicas, Universidad Nacional Autónoma de México, Mexico City, Mexico

**Keywords:** distal convoluted tubule, GABAergic activity, cell volume regulation, intracellular chloride concentration, arterial blood pressure, potassium

## Abstract

The role of Cl^–^ as an intracellular signaling ion has been increasingly recognized in recent years. One of the currently best described roles of Cl^–^ in signaling is the modulation of the With-No-Lysine (K) (WNK) – STE20-Proline Alanine rich Kinase (SPAK)/Oxidative Stress Responsive Kinase 1 (OSR1) – Cation-Coupled Cl^–^ Cotransporters (CCCs) cascade. Binding of a Cl^–^ anion to the active site of WNK kinases directly modulates their activity, promoting their inhibition. WNK activation due to Cl^–^ release from the binding site leads to phosphorylation and activation of SPAK/OSR1, which in turn phosphorylate the CCCs. Phosphorylation by WNKs-SPAK/OSR1 of the Na^+^-driven CCCs (mediating ions influx) promote their activation, whereas that of the K^+^-driven CCCs (mediating ions efflux) promote their inhibition. This results in net Cl^–^ influx and feedback inhibition of WNK kinases. A wide variety of alterations to this pathway have been recognized as the cause of several human diseases, with manifestations in different systems. The understanding of WNK kinases as Cl^–^ sensitive proteins has allowed us to better understand the mechanistic details of regulatory processes involved in diverse physiological phenomena that are reviewed here. These include cell volume regulation, potassium sensing and intracellular signaling in the renal distal convoluted tubule, and regulation of the neuronal response to the neurotransmitter GABA.

## Chloride as a Signaling Ion

The chloride (Cl^–^) anion is an important component of all known living beings, where it plays several roles in homeostatic and rheostatic processes in all types of cells. In humans, extracellular Cl^–^ concentration is maintained relatively constant, between 100 and 116 mmol/L, due to tight regulation by the kidneys and intestine (Boulpaep and Boron, 2016). It is notable that there is interspecies variability of plasma Cl^–^ concentration. For instance, normal levels in rats and mice are in the range of 90–132 mmol/L and 106–131 mmol/L, respectively (Lea et al., 2018).

Intracellular Cl^–^ concentration ([Cl^–^]_i_) varies wildly among different cell types within an organism. For example, it has been reported that most adult neurons have relatively low [Cl^–^]_i_ (5–15 mmol/L) (Kakazu et al., 1999; Yamada et al., 2004; Glykys et al., 2014), and [Cl^–^]_i_ of renal epithelial cells such as the ones of the distal convoluted tubule (DCT) has been estimated to be between 10 and 20 mmol/L (Beck et al., 1988; Boettger et al., 2002; Weinstein, 2005; Terker et al., 2015b). Conversely, olfactory sensory neurons (Reuter et al., 1998) and some cells from secretory epithelia, such as pancreatic (O’Doherty and Stark, 1983) and salivary acinar cells (Foskett, 1990), have a [Cl^–^]_i_ as high as 60–65 mmol/L. Additionally, [Cl^–^]_i_ can be dynamically modulated by different stimuli, such as cholinergic agonists (Foskett, 1990), cAMP levels (Xie and Schafer, 2004), lectin-stimulation (Lai et al., 2003), and extracellular potassium concentration ([K^+^]_e_) (Terker et al., 2015b). These reports exemplify the wide variation of [Cl^–^]_i_, which is important for the role that this anion plays in the physiology of specific cell types.

While some of the most studied roles for Cl^–^ in physiology are related to cell volume regulation (Hoffmann et al., 2009), establishment of resting membrane potential (Funabashi et al., 2010; Hutter, 2017), and acid-base balance (Seifter and Chang, 2016), it is now becoming clear that this anion is involved in intracellular signaling pathways involved in the regulation of a wider variety of cellular processes, such as gene expression, cell proliferation, apoptosis, among others (reviewed in Valdivieso and Santa-Coloma, 2019; Wilson and Mongin, 2019; Lüscher et al., 2020). For instance, published evidence suggests that [Cl^–^]_i_ can modulate the activity of different kinases, such as the MAPKs p38, JNK and ERK (Ohsawa et al., 2010; Wu et al., 2016), as well as SGK1 (Zhang et al., 2018), although it is still unclear whether direct effects of Cl^–^ ions on the kinases themselves are responsible. However, the With-No-lysine (K) (WNK) family of kinases is one example where the direct Cl^–^ binding to the enzyme’s active site (Piala et al., 2014) that modulates kinase activity (Bazua-Valenti et al., 2015) has been thoroughly studied (detailed in sections below). These observations support the novel proposed role of [Cl^–^]_i_ as a second messenger (Valdivieso and Santa-Coloma, 2019; Wilson and Mongin, 2019; Lüscher et al., 2020), responsible for modulating the activity of several proteins.

Transmembrane transport proteins determine the Cl^–^ permeability of each cell type. Ion channels that facilitate large Cl^–^ fluxes across cell membranes include the CLC family (reviewed extensively in Jentsch and Pusch, 2018), the cystic fibrosis transmembrane conductance regulator (CFTR) channel (Csanády et al., 2019), the volume-regulated anion channel (VRAC) channel (Osei-Owusu et al., 2018), and Ca^2+^-activated Cl^–^ channels (CaCCs) such as anoctamins (Pedemonte and Galietta, 2014). The direction of Cl^–^ flux through ion channels is solely determined by the electrochemical gradient of this ion across the membrane. However, secondary active transporters can set the [Cl^–^]_i_ at levels that diverge from the electrochemical equilibrium by coupling Na^+^ influx or K^+^ efflux to the movement of Cl^–^. Transporters with this type of activity are all members of the SLC12 family of solute carriers described below.

## The SLC12 Family of Cotransporters

The SLC12 family of solute carriers is comprised by the electroneutral cation-coupled Cl^–^ cotransporters (CCCs). Seven members of this family are arranged in two branches, depending on their ability to use Na^+^ as one of the transported cations coupled to Cl^–^. The Na^+^-dependent branch includes the Na^+^-K^+^-2Cl^–^ cotransporters, known as NKCC1 and NKCC2, and the Na^+^-Cl^–^ cotransporter, NCC (Gamba, 2005). NKCC1 is expressed in many epithelial and non-epithelial cells (Delpire et al., 1994). Within epithelial cells it is expressed in the basolateral membrane, except in the choroid plexus of the brain, where it is expressed apically (Delpire et al., 1994; Wu et al., 1998). NKCC2 is exclusively expressed in the apical membrane of the thick ascending limb of Henle’s loop in the kidney (Gamba et al., 1994) and NCC is present in the apical membrane of the distal convoluted tubule in the kidney and in osteoblasts in bone (Gamba et al., 1994; Dvorak et al., 2007). Identity degree at the amino acid level among these transporters is between 50 and 60%. The Na^+^-independent branch is composed of four K^+^-Cl^–^ cotransporters, known as KCC1 to KCC4. Of these four, KCC2 is exclusively expressed in neurons, while the other three KCCs are present in many cells throughout the body. Identity degree among KCCs is about 60% and between the Na^+^- dependent and independent branches is around 25% (Gamba, 2005; Arroyo et al., 2013).

The CCCs are secondary active transporters whose activity is driven by the Na^+^ and K^+^ gradients generated by the Na^+^-K^+^-ATPase. The Na^+^-driven transporters move ions from the extracellular space into the cytoplasm, while the K^+^-driven transporters (of the Na^+^-independent branch) mediate ion extrusion from the cells. In non-epithelial cells, the sustained activity of the Na^+^-K^+^-ATPase maintains a low Na^+^ and high K^+^ intracellular concentration, respectively. Thus, it is considered that the net effect of the activity of CCCs is the modulation of the [Cl^–^]_i_. Because of this, the expression of Na^+^-driven and K^+^-driven members of the SLC12 family in the same cell constitutes a system for the dynamic modulation of [Cl^–^]_i_. This, for example, is particularly relevant in neurons, where, as explained below in detail, the type and magnitude of the response to neurotransmitters that activate Cl^–^ channels in the postsynaptic membrane depends on the electrochemical Cl^–^ gradient (Kahle et al., 2008). Regulation of [Cl^–^]_i_ also plays a relevant role for the regulation of cell volume (de los Heros et al., 2018). In epithelial cells, the CCCs works in conjunction with other apical and/or basolateral channels and transporters to carry out transepithelial ion transport (Gamba, 2005). Thus, the major physiological roles of the CCCs are modulation of [Cl^–^]_i_, cell volume regulation, and transepithelial ion transport. For this reason, the CCCs are implicated in many organs’ and systems’ physiological processes (Gamba, 2005).

A variety of human and animal diseases and phenotypes in knockout mice models have been helpful in revealing the many roles of CCCs in physiology (Delpire and Mount, 2002) (Table 1). Inactivating mutations in the renal cotransporters NKCC2 and NCC are the cause of the Bartter syndrome type I (Simon et al., 1996a) and Gitelman disease (Simon et al., 1996b), respectively. NKCC2 constitutes the main apical entryway for Na^+^ and Cl^–^ in the thick ascending limb of Henle’s loop and NCC plays a similar role in the downstream adjacent nephron segment known as the distal convoluted tubule. Decreased Na^+^ reabsorption in these segments is not only associated with volume depletion and low blood pressure, but also with hypokalemic metabolic alkalosis due to increased K^+^ secretion in the aldosterone sensitive distal nephron that is stimulated by the increased distal Na^+^ delivery (Gamba, 2005). The phenotype of both, type I Bartter syndrome and Gitelman syndrome patients, is exclusively the consequence of the lack of activity of these transporters in the nephron, suggesting that indeed their expression is very restricted to the kidney and, if expressed elsewhere, like NCC in bone, its role in other tissues is not essential. Regarding NKCC1, knockout mice were generated and studied before human mutations in this gene were found. These mice have a wide variety of phenotypic alterations, such as small size, inner ear dysfunction, male infertility, altered pain perception, defects in intestinal transit, decreased saliva production, and low blood pressure, among others (Gagnon and Delpire, 2013). Delpire et al. (2016) described a human patient with respiratory weakness, endocrine and pancreatic abnormalities, and multi-organ failure. Genetic analysis revealed a heterozygous 11-bp deletion in exon 22 of *SLC12A2* (encoding NKCC1) (Delpire et al., 2016) that causes a frameshift resulting in a truncated protein lacking the last 187 amino acid residues of the C-terminus. This mutation causes the mislocalization of the cotransporter to the apical membrane in epithelial cells (Koumangoye et al., 2018). The same mutation has been shown to cause a similar, although milder, phenotype in mice (Koumangoye et al., 2020). Macnamara et al. (2019) reported a novel syndrome, named Kilquist syndrome, in a patient harboring a large homozygous deletion in *SLC12A2*, from intron 1 through exon 7. Such mutation leads to aberrant splicing between exons 1 and 8, introducing a frameshift that would produce a truncated protein. Molecular analysis showed lower mRNA levels and absence of the NKCC1 protein in the patient’s fibroblasts. Phenotypic features similar to the ones observed in NKCC1 knockout (−/−) mice were reported, including global developmental delay, bilateral sensorineural hearing loss, gastrointestinal abnormalities, and xerostomia (Macnamara et al., 2019). In addition, a missense variant that increases the activity of NKCC1 was described to be associated with schizophrenia (Merner et al., 2016).

**TABLE 1:** SLC12 cotransporters: associated genetic diseases and phenotype of knockout models.

Gene	Protein	Tissue expression	Associated disease (inheritance pattern; effect on protein function)	Knockout mice phenotype
*SLC12A1*	NKCC2	Kidney (Gamba et al., 1994); gastrointestinal tract (Xue et al., 2009)	**Bartter syndrome type I** (OMIM 601678) – hypokalemic metabolic alkalosis, low blood pressure, hypercalciuria (autosomal recessive; loss of function) (Simon et al., 1996a) **Hydrallantois** in *Bos taurus* (OMIA 002053-9913) – excessive accumulation of fluid within the allantoic cavity in pregnant animals (autosomal recessive; loss of function) (Sasaki et al., 2016)	**Bartter-like** – severe volume depletion and failure to thrive; partial rescue with indomethacin, with severe polyuria, hydronephrosis, hypokalemia, hypercalciuria, hyperreninemia, and proteinuria (Takahashi et al., 2000)
*SLC12A2*	NKCC1	Ubiquitous (Delpire et al., 1994)	**Multisystem dysfunction** – endocrine abnormalities, low blood pressure, intestinal dysfunction (autosomal dominant – mistrafficking of mutant NKCC1 to apical membrane in epithelial cells) (Delpire et al., 2016; Koumangoye et al., 2018) **Kilquist syndrome** – sensorineural deafness, intestinal and respiratory dysfunction, neuropsychological delay, severe xerostomia (autosomal recessive; loss of function) (Macnamara et al., 2019) **Schizofrenia** – (autosomal dominant; gain of function) (Merner et al., 2016)	Sensorineural deafness (Delpire et al., 1999; Dixon et al., 1999), growth retardation, low blood pressure, intestinal abnormalities (Flagella et al., 1999), male infertility (Pace et al., 2000), decreased saliva secretion (Evans et al., 2000), altered pain perception (Sung et al., 2000)
*SLC12A3*	NCC	Kidney (Gamba et al., 1994), bone (Dvorak et al., 2007), placenta, prostate and small intestine (Chang et al., 1996)	**Gitelman syndrome** (OMIM 263800) – hypokalemic metabolic alkalosis, low blood pressure, hypocalciuria, hypomagnesemia (autosomal recessive; loss of function) (Simon et al., 1996b)	**Gitelman-like** – low blood pressure in low Na^+^ diet, hypocalciuria, hypomagnesemia (Schultheis et al., 1998), metabolic alkalosis (Loffing et al., 2004), hypokalemia while in low K^+^ diet (Morris et al., 2006)
*SLC12A4*	KCC1	Ubiquitous (Gillen and Forbush, 1999)	None found	No obvious differences compared to littermate WT mice (Rust et al., 2007)
*SLC12A5*	KCC2	Central nervous system (Payne et al., 1996), pancreas (Kursan et al., 2017)	**Idiopathic epilepsy** (OMIM 616685) – (autosomal dominant with incomplete penetrance; loss of function) (Kahle et al., 2014; Puskarjov et al., 2014) **Epilepsy of infancy with migrating focal seizures** (OMIM 616645) – (autosomal recessive; loss of function) (Stödberg et al., 2015)	Neonatal death due to inability to breathe because of irregular activity of pre-Bötzinger complex (Hubner et al., 2001)
*SLC12A6*	KCC3	Wide, including muscle, heart, kidney, lung, and brain (Mount et al., 1999).	**Andermann syndrome** (OMIM 218000) – peripheral neuropathy associated with agenesis of the corpus callosum (autosomal recessive; loss of function) (Howard et al., 2002) **Spinocerebellar ataxia** in *Canis lupus familiaris* (OMIA 002279-9615) – (autosomal recessive; loss of function) (Van Poucke et al., 2019)	Locomotor abnormalities, deficit in prepulse inhibition, hypomyelination, axonal swelling fiber degeneration (Howard et al., 2002), progressive hearing loss, arterial hypertension, defective cell volume regulation (Boettger et al., 2003)
*SLC12A7*	KCC4	Wide, including heart, lung, liver, kidney, pancreas, stomach, thyroid (Mount et al., 1999)	None found	Deafness, renal tubular acidosis (Boettger et al., 2002)

Mutations in KCC2 have been implicated in a variety of epileptic syndromes (Kahle et al., 2014; Puskarjov et al., 2014) and mutations in KCC3 are the cause of a very complex inherited neurological disease known as Andermann’s syndrome in which patients exhibit absence of the corpus callosum in the brain, together with a variety of neurodegenerative and psychiatric manifestations (Howard et al., 2002). No pathogenic mutations in KCC4 have been described in humans. However, KCC4^–/–^ mice display deafness and renal tubular acidosis, suggesting that this transporter plays an important role in inner ear and kidney physiology (Boettger et al., 2002). Finally, global, constitutive disruption of KCC1 in mice has no phenotypic consequences, suggesting that the absence of KCC1 can be compensated by other K^+^-Cl^–^ cotransporters (Rust et al., 2007).

The Na^+^-driven and K^+^-driven cotransporters are regulated in opposite ways. They are all regulated by a kinase cascade in which the With No lysine (K) kinases (WNKs) phosphorylate and regulate the STE20-Proline Alanine rich Kinase (SPAK), and the Oxidative Stress Responsive Kinase 1 (OSR1). SPAK and OSR1 in turn phosphorylate the CCCs (Richardson and Alessi, 2008; Gagnon and Delpire, 2012; Alessi et al., 2014). Phosphorylation of the Na^+^-driven cotransporters, which occur in a cluster of serine-threonine residues located in the intracellular N-terminal domain, results in upregulation of cotransporter activity. In contrast, phosphorylation of K^+^-driven transporters that occurs in threonine residues of the C-terminal cytoplasmic domain results in downregulation of cotransporter activity. Thus, activation of this kinase cascade is able to simultaneously activate the Na^+^-(K^+^)-Cl^–^ influx and prevent the K^+^-Cl^–^ efflux, increasing [Cl^–^]_i_ and intracellular osmolarity, while inactivation of the phosphorylating cascade and/or activation of dephosphorylating pathways result in opposite effects.

Stimuli such as the decrease in [Cl^–^]_i_ or the decrease in cell volume (cell shrinkage) result in activation of the WNK-SPAK/OSR1 phosphorylation pathway, increasing the activity of the Na^+^-(K^+^)-Cl^–^ cotransporters and inhibiting the K^+^-Cl^–^ cotransporters (Gagnon et al., 2006; Zagórska et al., 2007; Arroyo et al., 2013; Piala et al., 2014; Bazua-Valenti et al., 2015; de los Heros et al., 2018). This results in the increase of [Cl^–^]_i_ or in the net influx of ions that contribute to the regulatory volume increase response. In contrast, an increase in [Cl^–^]_i_ or cell volume (cell swelling) inhibits the phosphorylating pathway and activates protein phosphatases, thus resulting in cotransporters dephosphorylation, and the consequent decrease in [Cl^–^]_i_ or the net efflux of ions that contribute to the regulatory volume decrease response. Thus, a negative feedback loop integrated by the monovalent cation-Cl^–^ cotransporters of the CCC family, the WNK-SPAK/OSR1 kinase cascade, and protein phosphatases serve to regulate the [Cl^–^]_i_ and/or the cell volume, which in turn modulate the activity of the cotransporters via the kinases and phosphatases. This feedback loop has implications in several physiological processes that are discussed in this work.

## The WNK-SPAK/OSR1 Signaling Pathway and Its Modulation by Intracellular Chloride

**FIGURE 1:**
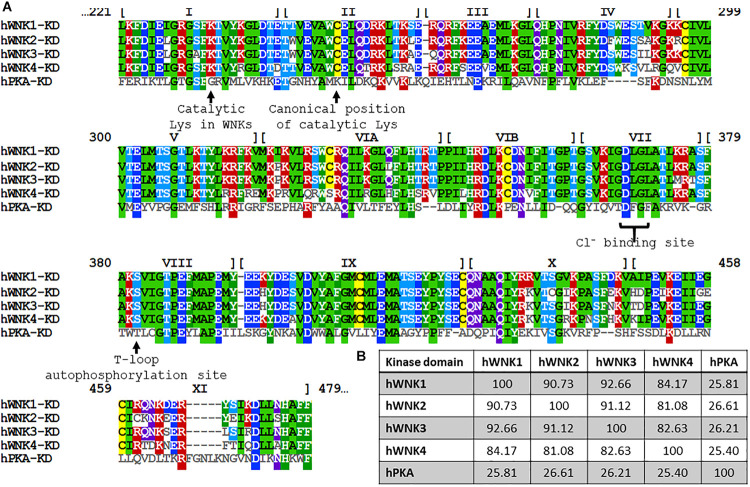
Alignment of the primary structure of kinase domains of human WNKs and the PKA. **(A)** Amino acid sequence alignment of the kinase domains of human WNK1 (UniProt accession number:Q9H4A3), WNK2 (Q9Y3S1), WNK3 (Q9BYP7), WNK4 (Q96J92) and PKA (P17612). Numbers at the top represent the residue numbers of WNK1. The roman numerals indicate the subdomains within the kinase domain. PKA was included for reference and comparison of functional residues, such as the positioning of catalytic Lys and critical residues involved in Cl^–^ binding in WNKs. **(B)** Percentage identity among kinase domains is significantly higher than identity among full-length proteins (see Figure 2). Notably, WNK4’s kinase domain is the most divergent one. Alignment and percentage identity matrix were generated in Clustal Omega (EMBL-EBI).

**FIGURE 2:**
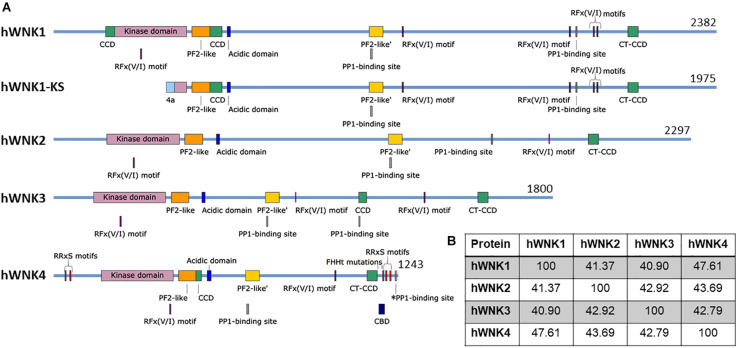
Schematic representation of human WNK kinases. **(A)** Four genes encoding WNK kinases exist in humans. An alternative promoter gives rise to the kinase-deficient kidney-specific (KS) isoform of WNK1 (Delaloy et al., 2003). Several confirmed and putative domains and binding sites are indicated, such as the kinase domain [Pfam (EMBL-EBI)] (El-Gebali et al., 2019), the PF2-like [Pfam (EMBL-EBI)] and PF2-like’ domains [similar to PF2 (PASK/Fray 2) domains in SPAK and OSR1] (Gagnon and Delpire, 2012), the acidic domain (responsible for interaction with KLHL3 and therefore, WNK degradation) (Ohta et al., 2013), RFx(V/I) motifs which mediate interaction with SPAK/OSR1 (Villa et al., 2007), predicted PP1-binding sites (RVxF motifs) (ELM, Kumar et al., 2020) and a confirmed PP1-binding site in WNK4 (*PP1) (Murillo-de-Ozores et al., 2018), the coiled-coil domains (CCD) (predicted by PCOILS, Gruber et al., 2006), including the C-terminal CCD (CT-CCD) mediating WNK-WNK interaction (Thastrup et al., 2012), the CaM-binding domain (CBD, studied in WNK4 but conserved in other WNKs) (Na et al., 2012), and the RRxS motifs that are phosphorylated by PKC/PKA and/or SGK1 in WNK4 (Rozansky et al., 2009; Na et al., 2012; Castañeda-Bueno et al., 2017). All reported FHHt mutations in WNK4 are located in the acidic domain, with the exception of K1169E (Zhang et al., 2011) and R1185C (Wilson et al., 2001) which are located in its C-terminus, while WNK1 FHHt mutations are intronic deletions that affect gene expression. It is noteworthy that alternative splicing is responsible for producing several isoforms of WNK3 and WNK1 (Holden et al., 2004; Vidal-Petiot et al., 2012), while proteolytic processing produces C-terminally-truncated WNK4 proteins (Murillo-de-Ozores et al., 2018). Finally, it is important to emphasize that some of these sites are based on prediction and experimental evidence will be necessary to assess their particular role. Figures were made in SnapGene software (from Insightful Science; available at snapgene.com). **(B)** Percent identity among WNK kinases shows the highest similarity between WNK1 and WNK4. Percentage identity matrix was generated in Clustal Omega (EMBL-EBI) (Sievers et al., 2011).

The WNK family of Ser/Thr kinases is comprised of four members in mammals: WNK1, WNK2, WNK3, and WNK4. Unlike most kinases, WNKs have their conserved catalytic Lys residue involved in ATP binding located in subdomain I, instead of in subdomain II, a characteristic that earned them their name (Xu et al., 2000) (Figure 1). Structurally, WNK kinases are composed by a relatively small regulatory N-terminal domain, followed by a highly conserved kinase domain (divided in the 12 subdomains characteristic of Ser/Thr kinases), and a large C-terminal domain with regulatory functions conferred by a variety of domains and binding sites for different interacting proteins (McCormick and Ellison, 2011; Gagnon and Delpire, 2012) (Figure 2). WNK1, WNK3, and WNK4 are expressed in a wide variety of tissues, such as heart, brain, lung, liver, muscle, kidney, testis, and colon, among others (Xu et al., 2000; Holden et al., 2004; Kahle et al., 2004; Vitari et al., 2005; Murillo-de-Ozores et al., 2018), while WNK2 is only expressed in brain, heart, and colon (Veríssimo and Jordan, 2001) (Table 2).

**TABLE 2:** Components of the WNK-SPAK/OSR1 pathway: associated genetic diseases, and phenotype of knockout models.

Gene	Protein	Tissue expression	Associated disease (inheritance pattern; effect on protein function)	Knockout mice phenotype
*STK39*	SPAK	Wide, including brain, adrenal gland, thymus, spleen, intestine, heart, kidney, testis, ovary, lung (Ushiro et al., 1998)	None found	**Gitelman-like** – low blood pressure, hypokalemia, hypocalciuria, hypomagnesemia, decreased NCC activity (Yang et al., 2010)
*OXSR1*	OSR1	Ubiquitous (Tamari et al., 1999)	None found	Early embryonic lethality, similar to WNK1^–/–^ mice (Xie et al., 2013)
*WNK1*	WNK1	Wide, including kidney, testis, heart, brain, spleen, muscle, lung, liver, pancreas, adipose tissue (Xu et al., 2000; Vitari et al., 2005)	**Familial Hyperkalemic Hypertension (FHHt)** (OMIM 614492) – hyperkalemia, hypertension, metabolic acidosis (autosomal dominant; gain of function) (Wilson et al., 2001) **Hereditary Sensory and Autonomic Neuropathy type IIA (HSAN2A)** (OMIM 201300) – reduced sensation to pain, temperature, and touch (autosomal recessive; loss of function) (Shekarabi et al., 2008)	Early embryonic lethality (Zambrowicz et al., 2003) due to cardiovascular developmental and placental defects (Xie et al., 2009)
*WNK2*	WNK2	Brain, heart and colon (Veríssimo and Jordan, 2001)	None found	Not reported yet
*WNK3*	WNK3	Wide, including kidney, colon, heart, brain, muscle, lung, liver, pancreas, placenta (Holden et al., 2004)	None found	No obvious differences to littermate WT mice in basal conditions, low blood pressure in low Na^+^ diet (Oi et al., 2012)
*WNK4*	WNK4	Wide, including kidney, testis, colon, heart, brain, spleen, lung, liver (Kahle et al., 2004; Murillo-de-Ozores et al., 2018)	**Familial Hyperkalemic Hypertension (FHHt)** (OMIM 614491) – hyperkalemia, hypertension, metabolic acidosis (autosomal dominant; gain of function) (Wilson et al., 2001) **Hypokalemic periodic paralysis** in *Felis catus* (OMIA 001759-9685) – skeletal muscle weakness with intermittent hypokalemia (autosomal recessive; loss of function) (Gandolfi et al., 2012)	**Gitelman-like** – normal blood pressure with increased renin activity, hypokalemic metabolic alkalosis, hypomagnesemia (Castaneda-Bueno et al., 2012)

The first evidence linking WNK kinases to the CCCs was the discovery of mutations in the genes *WNK1* and *WNK4* in Familial Hyperkalemic Hypertension (FHHt), also known as Pseudohypoaldosteronism type 2 (PHAII), or Gordon syndrome (Wilson et al., 2001). FHHt is mainly driven by overactivation of NCC in the distal nephron (Lalioti et al., 2006; Grimm et al., 2017). Further studies showed that WNKs activate N(K)CCs and inhibit KCCs by phosphorylating and activating the downstream kinases SPAK and OSR1 (Moriguchi et al., 2005; Vitari et al., 2005; Alessi et al., 2014).

SPAK and OSR1 are two highly similar Ser/Thr kinases of the Ste20 family, that display wide tissue expression (extensively reviewed by Gagnon and Delpire, 2013) (Table 2). Initially, these kinases were shown to bind the CCCs by a yeast two-hybrid screen (Piechotta et al., 2002) and it was later shown that they are responsible for direct phosphorylation of the CCCs (Dowd and Forbush, 2003; de los Heros et al., 2014). SPAK and OSR1 contain a domain called PF2 in their C-terminal region (also called CCT), that mediates binding with RFx(V/I) motifs in WNK kinases (Villa et al., 2007) and in CCCs (Piechotta et al., 2002). Accordingly, mice with a mutation in SPAK PF2 domain (L502A) display lower SPAK and CCCs phosphorylation levels (Zhang J. et al., 2015). Interestingly, it has been proposed that two regions with a similar tertiary structure exist in WNK kinases themselves (PF2-like and PF2-like’) (Gagnon and Delpire, 2013). Mutation of PF2-like’ in WNK4 prevents SPAK phosphorylation (Murillo-de-Ozores et al., 2018), and although the role of these regions is currently unknown, they might play a role in WNK binding with each other and/or with the CCCs (Moon et al., 2013).

### WNKs as Cl^–^-Sensing Kinases

The role of WNK kinases as Cl^–^ sensing proteins was suggested since their initial characterization. It was shown that the reduction in [Cl^–^]_i_ induced kinase autophosphorylation and activation (Moriguchi et al., 2005). Increased activation and phosphorylation of NKCC1, NKCC2, and NCC by lowering [Cl^–^]_i_ also suggested that WNK kinases were likely modulated by [Cl^–^]_i_ (Breitwieser et al., 1990; Lytle and Forbush, 1996; Pacheco-Alvarez et al., 2006; Ponce-Coria et al., 2008).

Conclusive evidence that WNK kinases are Cl^–^−sensitive proteins came from X-ray crystallography studies performed by Piala et al. (2014). Crystallographic structure of the kinase domain of rat WNK1 showed the presence of a Cl^–^ anion bound directly to the kinase, specifically to the backbone amides of Gly370 and Leu371, located in the N-terminus of the activation loop, with additional hydrophobic interactions with Phe283, Leu299, Leu369, and Leu371 (a Cl^–^ binding pocket structurally similar to the one observed in ClC transporters). Direct Cl^–^ binding stabilizes a WNK1 inactive conformation, while decreased [Cl^–^] or mutation of the Cl^–^ binding site, by substituting Leu369 for a Phe (L369F), resulted in increased autophosphorylation and activation of WNK1 kinase. Interestingly, as the Cl^–^ binding site is located in the catalytic site of the kinase, it might overlap with the canonical positioning of the catalytic Lys in other kinases. Thus, the unique placement of this Lys in WNKs in subdomain I might permit Cl^–^ binding. Cl^–^-sensitivity of WNK kinases seems to be a conserved regulatory mechanism, as it has been shown that Cl^–^ also inhibits *Drosophila melanogaster* WNK (DmWNK) autophosphorylation (Sun et al., 2018).

Reports of WNK4 effect over NCC activity were initially discordant, because evidence showed inhibitory modulation *in vitro* (Wilson et al., 2003; Yang et al., 2003), while *in* vivo evidence pointed to WNK4 as an activator of NCC (Castaneda-Bueno et al., 2012). Later, it was described that this discrepancy could be explained by WNK4 regulation by [Cl^–^]_i_. Bazua-Valenti et al. (2015) showed that while WNK4 coexpression does not upregulate NCC in basal conditions in *Xenopus laevis* oocytes, decreasing [Cl^–^]_i_ promotes WNK4’s activating phosphorylation and WNK4-mediated NCC activation. Mutation of the WNK4 Cl^–^-binding site (L322F in human WNK4) turned it into a constitutively active kinase that upregulated NCC activity, even without Cl^–^ depletion. These series of experiments not only helped to understand the different effects of WNK4 over NCC function, but also confirmed WNK kinases as key Cl^–^ sensing proteins that regulate the activity of the CCCs.

Analysis of WNK1, WNK3, and WNK4 autophosphorylation upon Cl^–^ depletion in *X. laevis* oocytes (Bazua-Valenti et al., 2015), as well as *in vitro* kinase assays (Terker et al., 2015a) have suggested different Cl^–^ sensitivities for these three kinases. While WNK1 and WNK4 autophosphorylation was increased by incubating oocytes in a hypotonic low Cl^–^ media, WNK3 phosphorylation was not affected by this maneuver as it was already phosphorylated in basal conditions (Bazua-Valenti et al., 2015). *In vitro* kinase assays, incubating the recombinant kinase domains of WNK1, WNK3, or WNK4 with their substrate SPAK in buffers with different [Cl^–^], showed that WNK4 was inhibited in a lower range of [Cl^–^]s than WNK1 or WNK3 (Terker et al., 2015a). Coexpression of NCC with Cl^–^-insensitive mutants of WNK1 (L369F/L371F) and WNK4 (L322F/L324F) dramatically increased NCC phosphorylation when compared to pNCC levels in the presence of their wild type (WT) counterparts. However, WNK3 L295F/L297F did not affect NCC differently from WT WNK3 as this kinase is already active even in cells with high [Cl^–^]_i_ (∼70 mM in HEK cells and ∼55 mM in oocytes) (Bazua-Valenti et al., 2015; Terker et al., 2015a; Pacheco-Alvarez et al., 2020). These studies suggested that WNK3 activity is independent of [Cl^–^]_i_. Thus, although WNK3 can modify [Cl^–^]_i_ through the regulation of the CCCs, [Cl^–^]_i_ is not the main regulator of WNK3 activity, as we discuss below.

It is worth mentioning that there are some differences in the specific values of [Cl^–^] that inhibit WNK activity (Piala et al., 2014; Terker et al., 2015a; Sun et al., 2018). Discrepancies could be due to the use of different substrates’ phosphorylation as a surrogate for WNK activity (such as myelin basic protein, OSR1, SPAK, or WNK itself). Additionally, *in vitro* assays have been performed using only the kinase domain of WNK kinases, while it is possible that the N- and C-terminal regions could affect Cl^–^ sensitivity. However, *in vitro* experiments have served as proof of concept to demonstrate that the kinase activity of WNKs can be directly modulated by Cl^–^ binding. This phenomenon has more recently been corroborated *in vivo* in flies expressing a Cl^–^ insensitive DmWNK (L421F). DmWNK regulates K^+^-flux in the fly’s Malpighian tubule and the DmWNK-L421F is more active than its WT counterpart (Sun et al., 2018). Moreover, Chen and collaborators generated mice with a Cl^–^ insensitive WNK4 (L319F/L321F). These mice display an altered renal phenotype, reminiscent of FHHt, as shall be explained in a later section, showing that WNK4 is indeed a physiological Cl^–^ sensitive protein (Chen et al., 2019).

These recent findings related to WNKs regulation by [Cl^–^]_i_ are helping to elucidate how the WNK-SPAK/OSR1-CCCs signaling pathway is involved in diverse physiological processes such as regulation of cell volume (Pacheco-Alvarez et al., 2020), potassium sensing and signaling in the renal distal convoluted tubule (Terker et al., 2015b), and differential neuronal response to GABA (Alessi et al., 2014). These processes will be discussed in the following sections.

## Role of the WNK-SPAK/OSR1-CCC Pathway in Cell Volume Regulation

Excluding bacterial and plant cells, all other cells are challenged constantly by changes in their volume due to differences in osmotic pressures between the intracellular and the extracellular milieu. Cell volume changes are proportional to the osmotic challenge they face. Water diffuses from the least concentrated solution to the more concentrated one, to equilibrate the osmotic pressure, causing cells to swell or shrink as osmotic balance is restored. However, basal cell volume must be promptly restored to minimize disruption of cellular functions and organization (Hoffmann et al., 2009).

**FIGURE 3:**
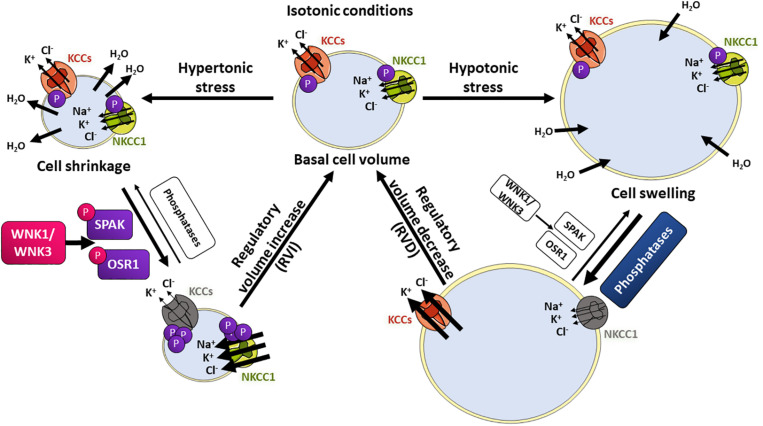
The WNK/SPAK/CCC signaling pathway in cell volume homeostasis. Changes in extracellular osmolarity induce cell shrinkage or swelling. Hypertonic stress with higher extracellular solute concentrations will induce water loss and cell shrinkage. This activates the regulatory volume increase (RVI) response, in which influx of Na^+^, K^+^, and Cl^–^ ions is stimulated to restore normal cell volume. On the contrary, lower extracellular solute concentrations (hypotonic stress) will induce water molecules entry and swelling, activating the regulatory volume decrease (RVD) response, in which K^+^ and Cl^–^ efflux is stimulated, followed by water loss and cell volume restoration. Transport proteins involved in RVI include the NKCC1 (green) cotransporter that is phosphorylated and activated by the SPAK and OSR1 kinases, which in turn are activated by phosphorylation mediated by the volume-sensitive kinases WNK1 and WNK3. The KCC cotransporters (orange) are inhibited by WNK-SPAK/OSR1-mediated phosphorylation. During RVD, KCCs and NKCC1 are dephosphorylated by phosphatases, which results in net K^+^ and Cl^–^ efflux.

When cells are exposed to hypotonic conditions, the resulting cell swelling triggers the regulatory volume decrease (RVD) response (Figure 3). The early phase of RVD involves activation of ion transporters that mediate K^+^ and Cl^–^ efflux. Water molecules will follow these ions until cell volume is restored. On the contrary, when cells are exposed to hypertonic conditions, cell shrinkage triggers the regulatory volume increase (RVI) response, which involves intracellular solute accumulation causing the osmotic influx of water and the recovery to normal cell volume. Na^+^, K^+^, and Cl^–^ ions influx occurs in the early phase of this response (de los Heros et al., 2018; Delpire and Gagnon, 2018). Transport systems on the membrane are activated within seconds of volume deviation. Transport proteins involved in RVD include the K^+^-Cl^–^ cotransporters (KCCs), the volume-regulated Cl^–^ channel VRAC, as well as K^+^ channels. For RVI, the main molecular players involved are the Na^+^-H^+^ exchanger and the Na^+^-K^+^-2Cl^–^ cotransporter (NKCC1) (Koivusalo et al., 2009). In this section we will focus on the mechanisms for CCCs’ activation and deactivation during cell volume regulation.

As mentioned above, a key signaling event for modulation of CCCs in response to cell volume changes is the phosphorylation or dephosphorylation of specific residues in their N- and C-terminal tails. For instance, during cell swelling, dephosphorylation of conserved C-terminal threonine residues in KCCs induces their activation, while dephosphorylation of conserved N-terminal residues in NKCC1 inhibits its activity (Lytle and Forbush, 1992; Darman and Forbush, 2002; Rinehart et al., 2009). This results in increased K^+^-Cl^–^ efflux and decreased Na^+^ influx, promoting water to follow ion movement. In contrast, during cell shrinkage, increasing phosphorylation of the above-mentioned sites, results in NKCC1 activation and KCCs inhibition, and thus, increased Na^+^, K^+^, and Cl^–^ influx, with the consequent influx of water that follows. This reciprocal regulation has led to propose the existence of a volume-sensing enzyme cascade that regulates both NKCCs and KCCs in opposite ways. The identity of the sensors and transducers have been studied and examined, and the WNK-SPAK/OSR1 signaling cascade has emerged as the cascade involved in such regulation, with protein phosphatases playing also an essential regulatory role (de Los Heros et al., 2006; Zagórska et al., 2007; Pacheco-Alvarez et al., 2020).

### *In vitro* Evidence of WNK1’s Role in Cell Volume Regulation

Different groups have shown that WNK1 activity is stimulated by hypertonic stress. For instance, Xu et al. (2000) and Zagórska et al. (2007) showed that WNK1 immunoprecipitated from HEK293 cells grown in hypertonic media displayed higher levels of autophosphorylation and higher ability to phosphorylate OSR1, respectively. Hypertonic stress promoted WNK1 autophosphorylation at S382, the T-loop’s residue, an event that promotes kinase activation (Xu et al., 2002; Zagórska et al., 2007). Hypertonic stress has also been shown to promote activation of SPAK and OSR1 in cellular models (Chen et al., 2004; Anselmo et al., 2006; Zagórska et al., 2007). Such activation correlates with kinases’ phosphorylation at activating sites targeted by WNK kinases (T-loop and S-motif sites). Additionally, siRNA-mediated WNK1 depletion in HeLa cells has been shown to partially prevent sorbitol-induced phosphorylation and activation of SPAK and OSR1 (Zagórska et al., 2007), suggesting that WNK1 activation in these cells is at least partially responsible for SPAK/OSR1 activation.

Regarding the mechanisms implicated in the modulation of WNK1 activity by hyperosmotic stress, it is interesting to note that Zagórska et al. (2007) observed that the truncated 1-667 WNK1 protein retained the ability of becoming activated by this stimulus, suggesting that the domain or domains involved in kinase activation are located within this region. In a more recent work, Naguro et al. (2012) showed that the MAP3K apoptosis signal-regulated kinase 3 (ASK3) is an osmotic stress-sensitive protein that can bind WNK1 and regulate its T-loop’s phosphorylation. More specifically, they showed that ASK3 becomes phosphorylated and activated when stimulated by hypotonic stress and inhibited by hypertonic stress. They also showed that, when activated by hypotonicity, ASK3 inhibits WNK1 autophosphorylation, because in ASK3 depleted cells, but not in control cells, an increase in WNK1-mediated OSR1 phosphorylation was observed when the cells were subjected to hypotonic stimulation. Given that hypotonic stress promotes [Cl^–^]_i_ reduction, for example, via activation of the VRAC Cl^–^ channel, an appealing hypothesis is that ASK3 may be important for preventing Cl^–^ depletion-induced WNK1 activation under this condition.

### *In vitro* Evidence of WNK3’s Role in Cell Volume Regulation

As it was discussed previously, among the WNK family, WNK1 and WNK4 are sensitive to [Cl^–^]_i_, while WNK3 constitutes the non-Cl^–^-sensitive member of the family and it might function as a cell volume-sensitive kinase. Indeed, WNK3 has shown unique biochemical and functional regulatory properties over the CCCs that have led to propose this protein as possible candidate for the volume sensor kinase. Initial characterization of WNK3 in 2005 performed in *Xenopus laevis* oocytes showed that WNK3 is a potent activator of NKCC1, NKCC2, and NCC, and an inhibitor of KCCs. In contrast, a catalytically inactive version of WNK3 (WNK3-KD) had opposite effects, most likely due to dominant negative effects on the endogenous WNK-SPAK/OSR1 cascade (Kahle et al., 2005; Rinehart et al., 2005; de Los Heros et al., 2006). No other WNK kinase has been shown to possess the ability to regulate all N(K)CCs and KCCs in such manner. It was also shown that WNK3 regulation of KCCs function, depend on phosphatase activity. By using phosphatase inhibitors, protein phosphatase 1 and 2B were proposed to be involved in the signaling pathway.

Experiments performed in HEK293 cells showed that WNK3 has a direct effect on the RVD and RVI volume compensatory mechanisms in mammalian cells. Cruz-Rangel et al. (2012) observed that cells that overexpress WNK3, achieved through stable transfection, showed less efficient RVD, which correlated with lower KCC activity and decreased Cl^–^ efflux. In contrast, cells overexpressing WNK3-KD showed more efficient RVD, as well and higher KCC activity and Cl^–^ efflux. Later on, in 2016, Zhang and coworkers used a combination of screening methods (kinome wide siRNA-screens, kinase inhibitor screen, and a kinase trapping- Orbitrap mass spectrometry screen) that allowed them to identify the major endogenous WNK kinase responsible for stimulating basal KCC3 C-terminal phosphorylation (T991 and T1048) in HEK293 cells (Zhang et al., 2016). WNK3 was identified in all three screening methods, suggesting that WNK3 basal activity is high in these cells. Subsequent targeted knockout experiments in the same model showed that not only WNK3 knockout, but also WNK1 knockout decreased KCC3 and NKCC1 phosphorylation in this model. They showed that HEK293 cells expressing KCC3 and transiently transfected with wild type WNK3 swelled under hypotonic stimulation, while expression of WNK3-KD prevented hypotonic swelling. This effect of WNK3-KD overexpression was reversed in the presence of the KCCs inhibitor furosemide, revealing that increased KCC activity was responsible for the more efficient RVD response in these cells. Finally, treatment of cells expressing wild type WNK3 with the SPAK/OSR1 inhibitor STOCK1S-50699, also prevented cells from swelling under hypotonic stress, demonstrating that inhibition of the WNK3-SPAK/OSR1 pathway promoted RVD. These experiments established the WNK3-SPAK/OSR1 complex as an integral component of the Cl^–^/volume sensitive kinase system regulating the CCCs.

**FIGURE 4:**
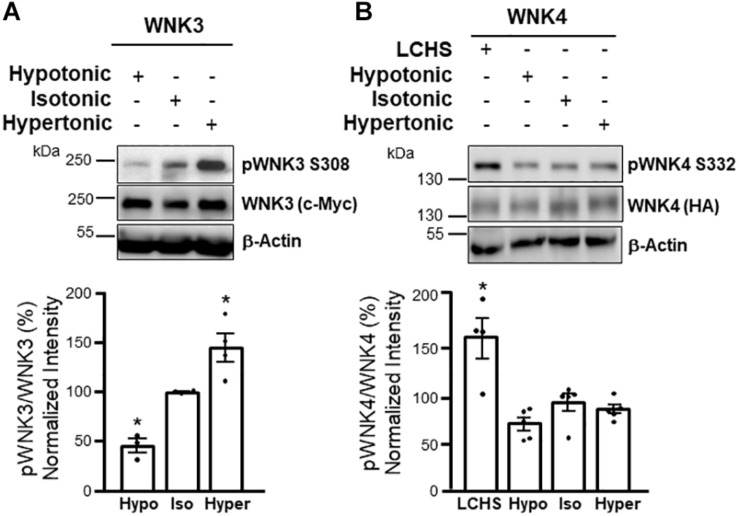
WNK3 phosphorylation is modulated by cell volume changes, while WNK4 phosphorylation is not altered by these stimuli, but responds to [Cl^–^]_i_ depletion. Western blot assays (upper panels) and corresponding results from densitometric analysis (lower panels) show that T-loop autophosphorylation of WNK3 (S308 in human WNK3) is decreased by incubation of oocytes in hypotonic media, while it is increased by incubation in hypertonic media **(A)**. In contrast, WNK4 T-loop autophosphorylation (S332 in mouse WNK4) is not affected by these maneuvers, but it is increased by low Cl^–^ hypotonic stress (LCHS) that promotes [Cl^–^]_i_ depletion **(B)**. These results suggest that WNK3 activity primarily responds to changes in cell volume, while WNK4 is mainly regulated by [Cl^–^]_i_. *p < 0.05 vs control. Modified from Pacheco-Alvarez et al. (2020) with permission.

More recently, using the *X. laevis* oocytes heterologous expression system we have shown that WNK3 activity towards NCC is not affected neither by changes in [Cl^–^]_i_, nor by eliminating the putative Cl^–^ binding sites on the kinase (Pacheco-Alvarez et al., 2020). Instead, the activating phosphorylation of WNK3’s T-loop, is modulated by changes in extracellular osmolarity (increased by hypertonicity and decreased by hypotonicity), whereas phosphorylation of WNK4 in the homologous residue is not affected by such stimuli (Figure 4). In contrast, WNK4 T-loop phosphorylation is stimulated by [Cl^–^]_i_ depletion. This supports the hypothesis that, at least toward the CCCs, while WNK4 and WNK1 are the Cl^–^ sensitive kinases, WNK3 is instead involved in volume-sensing. Key questions that remain open are whether this kinase can act as the actual cellular osmosensor or whether it is regulated instead by another protein with such activity. Also, whether WNK3 activity modulation by cell volume could also affect other players in the RVI or RVD response remains to be determined.

### The Activity of the WNK3-SPAK/OSR1-CCC Pathway Is Altered in Cerebral Edema in Rodent Models

*In vivo* model experiments analyzed the role of *the WNK3-SPAK/OSR1-CCC* complex in cerebral edema, a condition where volume homeostasis of brain cells is disrupted. It is worth mentioning that increased glial cell volume is the major contributor to cerebral edema, given that in the mammalian brain glia outnumber neurons and also because glia, unlike neurons, express aquaporins that render them more vulnerable to osmotic stress (Kahle et al., 2015). NKCC1 and KCC3 are highly expressed in astrocytes where they participate in cell volume regulation (Pearson et al., 2001; Su et al., 2002).

Middle cerebral artery occlusion (MCAO) was performed to produce brain ischemia, a maneuver that induces cerebral edema, in wild type (WT), WNK3^–/–^, and SPAK^–/–^ mice (Begum et al., 2015; Zhang et al., 2016; Zhao et al., 2017). In comparison to WT mice, WNK3^–/–^ and SPAK^–/–^ mice showed reduced cerebral edema and infarct volume after MCAO, as well as less demyelination and faster neurobehavioral recovery. Phosphorylation levels of KCC3 and NKCC1 in brain homogenates of WNK3^–/–^ mice after brain ischemia were decreased compared to those in wild type mice (Begum et al., 2015; Zhang et al., 2016). It was suggested that these effects on CCCs phosphorylation could account for the decreased cerebral edema and other improved outcomes. Supporting the key role of SPAK in the regulation of NKCC1, KCC2, and KCC3 activity in brain tissue, it was shown that SPAK-CCT domain knock-in mice (*SPAK^*L502A/L502A*^)*, in which SPAK is unable to bind CCCs, have lower levels of phosphorylated NKCC1, KCC2, and KCC3 in brain homogenates. Co-immunoprecipitation experiments of KCC3 with SPAK performed with brain lysates of these mice confirmed that the KCC3-SPAK interaction is disrupted. Therefore, these results place the WNK3-SPAK complex as a “volume sensor-transducer” in mammalian brain that regulates CCC activity to achieve volume homeostasis.

## Regulation of Renal Na-Cl Cotransporter (NCC) in Response to Changes in Extracellular K^+^ Concentration

The fine tuning of urinary Na^+^ and K^+^ excretion takes place within the mammalian distal nephron of which the distal convoluted tubule (DCT) is the very first segment. The DCT actively participates in Na^+^, Ca^2+^, and Mg^2+^ reabsorption, and thus, its activity has an impact on blood pressure, Ca^2+^ and Mg^2+^ homeostasis. NCC constitutes the apical entry pathway for Na^+^ and Cl^–^ to DCT cells and its activity is the rate-limiting step for NaCl reabsorption in this segment. In addition, even though no net K^+^ reabsorption or secretion occurs in the DCT, NCC activity has an important impact on renal K^+^ handling, and thus, renal K^+^ excretion (Subramanya and Ellison, 2014; Bazúa-Valenti et al., 2016). This is evidenced by the phenotype displayed by patients with Gitelman’s syndrome, a genetic disease caused by inactivating mutations in the *SLC12A3* gene that encodes NCC (Simon et al., 1996b). Patients present with hypotension, hypocalciuria, and hypomagnesemia, but also, one of the most notable features is renal K^+^ loss and hypokalemia. NCC activity affects renal K^+^ handling by indirectly affecting the activity of the secretory K^+^ apparatus of the aldosterone sensitive distal nephron (ASDN) comprised by the connecting tubule and the cortical collecting duct. In these segments aldosterone drives K^+^ secretion by stimulating the concerted action of apical epithelial Na^+^ channels (ENaC) and Renal Outer Medullary K^+^ channels (ROMK). Electrogenic Na^+^ reabsorption via ENaC generates a lumen-negative transepithelial potential that drives K^+^ secretion via ROMK. Big K^+^ (BK) channels are also important contributors to K^+^ secretion under certain conditions (Meneton et al., 2004; Murillo-de-Ozores et al., 2019). The mechanisms explaining NCC’s impact on K^+^ secretion by the ASDN are currently controversial. It was initially thought that, by affecting distal Na^+^ delivery, NCC activity could impact on ENaC’s activity, and thus K^+^ secretion. However, some recent works have failed to confirm this mechanism, and instead, recent data point out to a more complex interaction that involves remodeling of distal tubule segments (Hunter et al., 2014; Grimm et al., 2017).

Whichever the mechanism, the importance that NCC plays on modulation of renal K^+^ excretion is evidenced by the fact that physiological mechanisms exist to modulate NCC activity in response to changes in dietary K^+^ intake. NCC activity, assessed by measuring activating phosphorylation (Pacheco-Alvarez et al., 2006), increases in mouse models subjected to low K^+^ diets and decreases in mouse models subjected to high K^+^ diets (Vallon et al., 2009; Sorensen et al., 2013; Castaneda-Bueno et al., 2014; Terker et al., 2015a). When this mechanism is broken, alterations in K^+^ homeostasis occur, like it is observed in Gitelman’s syndrome, caused by loss of function of NCC, or in Familial Hyperkalemic Hypertension (FHHt), which appears to be mainly caused by overactivation of NCC (Wilson et al., 2001; Lalioti et al., 2006). As the disease name indicates, FHHt patients present hypertension with hyperkalemia, as well as hyperchloremic metabolic acidosis. FHHt-causative mutations do not occur in the *SLC12A3* gene, but in genes encoding proteins that participate in the regulation of NCC activity. These genes include, as previously discussed, two that encode the WNK kinases WNK1 and WNK4 (Wilson et al., 2001), and two more (*CUL3* and *KLHL3*) that encode components of a protein complex with ubiquitin ligase activity that regulate WNK1 and WNK4 ubiquitylation and degradation (Boyden et al., 2012; Louis-Dit-Picard et al., 2012; Ohta et al., 2013; Shibata et al., 2013; Wakabayashi et al., 2013).

**FIGURE 5:**
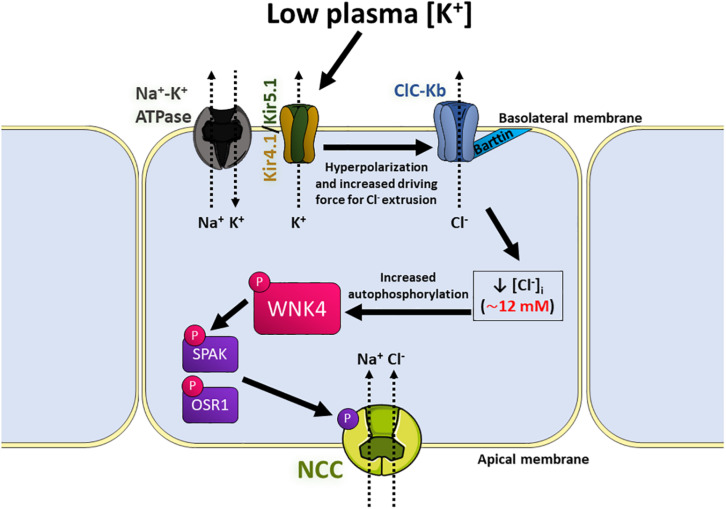
Model of the signaling pathway that activates NCC in response to low extracellular [K^+^] in the distal convoluted tubule (DCT). Low K^+^ intake can subtly decrease plasma [K^+^]. This upregulates the basolateral K^+^ conductance mediated by Kir4.1/5.1 heterotetramers located in the basolateral membrane of DCT cells, which causes hyperpolarization of membrane potential and increases the driving force for Cl^–^ efflux through the ClC-Kb channels, also located in the basolateral membrane by its association with its β-subunit, Barttin. This process lowers [Cl^–^]_i_, and therefore allows dissociation of a Cl^–^ anion from the kinase domain of WNK4, leading to kinase activation and autophosphorylation. Activated WNK4 then phosphorylates the kinases SPAK and OSR1, who phosphorylate and activate NCC, increasing NaCl reabsorption by these cells.

The regulation of [Cl^–^]_i_ in DCT cells is a key part of the signaling mechanism that mediates regulation of NCC in response to the subtle changes in extracellular K^+^ concentration ([K^+^]_e_) resulting, for example, from variations in dietary K^+^ content. As explained below in detail, expression of a specific subset of monovalent ion channels in the basolateral membrane of these cells allows translating changes of extracellular K^+^ levels into changes in membrane potential that in turn drive Cl^–^ fluxes that alter [Cl^–^]_i_ (Terker et al., 2015b). Such fluctuations in [Cl^–^]_i_ are sensed by the Cl^–^ sensitive WNK4, the master regulator of the signaling cascade involved in the regulation of NCC (Figure 5). In this section we describe the molecular players involved in this relatively novel signaling mechanism, as well as an overview of the *in vitro* and *in vivo* evidence that has been key for the description of this pathway.

### Direct Effects of Plasma K^+^ on DCT’s [Cl^–^]_i_ and NCC Activity

A decrease in NCC phosphorylation (pNCC) levels can be observed within 15 minutes after an acute K^+^ oral load. This effect parallels the rise in plasma K^+^ levels and precedes the rise in plasma aldosterone and activation of ENaC. Interestingly, the rapid natriuresis and kaliuresis induced by the K^+^ load seem to be dependent on the inhibition of NCC, because they are not observed in NCC^–/–^ mice (Sorensen et al., 2013). Decreased pNCC levels are also observed in mice in which hyperkalemia is induced pharmacologically by treatment with amiloride (Terker et al., 2015b) or in genetic mouse models with hyperkalemia, like in mice with specific deletion of ENaC subunits in the nephron (Perrier et al., 2016; Boscardin et al., 2017, 2018). Conversely, in rodent models with hypokalemia, e.g., in hyperaldosteronism models, NCC activation is observed (Kim et al., 1998; Terker et al., 2016). Finally, it has been shown by Terker et al. (2015a) that, across physiological levels of [K^+^]_e_, a linear correlation is observed between pNCC levels and [K^+^]_e_, and that subtle changes occur in [K^+^]_e_ in response to modest changes in dietary K^+^ content, that are responsible for variations in pNCC observed.

Terker et al. (2015b) based on experiments performed in HEK293 cells, proposed that changes in [K^+^]_e_ modulate pNCC by inducing changes in the [Cl^–^]_i_, that in turn affect the activity of the WNK-SPAK/OSR1 pathway. The physiological relevance of this model was supported by *ex vivo* experiments performed by Penton et al. (2016) in which isolated mice kidneys were perfused or mouse kidney slices were incubated with solutions containing variable [K^+^]. They confirmed that the [K^+^]_e_ has a direct effect on pNCC levels in the DCT. Moreover, such effects were not observed when changes in [Cl^–^]_i_ were prevented. Finally, they showed that whereas the activation of NCC on low [K^+^] is completely Cl^–^-dependent, Cl^–^-independent mechanisms also exist for the high [K^+^]-induced dephosphorylation of NCC.

### Kir4.1/5.1 Heterotetramers

Recent works support the idea that K^+^ channels formed by Kir4.1/5.1 heterotetramers are key for the direct sensing of extracellular [K^+^] by DCT cells. Inwardly rectifying K^+^ (Kir) channels are formed by homo- or hetero-tetramers, where each subunit is composed of two transmembrane regions with amino- and carboxyl-terminal regions located in the cytoplasm. These channels are expressed in a wide variety of cell types and are responsible for different functions (extensively reviewed by Hibino et al. (2010), one of which is the maintenance of resting membrane potential (Su and Wang, 2016).

In mouse microdissected DCT tubules, basolateral inwardly rectifying K^+^ channels identified as Kir4.1/5.1 heterotetramers were characterized by patch-clamp experiments (Lourdel et al., 2002; Zhang et al., 2014), and the expression of these channels was confirmed by RT-PCR (Lourdel et al., 2002). Immunostaining assays have also shown basolateral localization of Kir4.1 (Bockenhauer et al., 2009; Zhang et al., 2014; Cuevas et al., 2017) and Kir5.1 (Tucker et al., 2000; Zhang C. et al., 2015) in the DCT, and proteomic data from microdissected tubules from rats shows high levels of Kir4.1 and Kir5.1 in the DCT (Limbutara et al., 2020). Coincidentally, in vivo interaction between Kir4.1 and Kir5.1 was first found in kidney samples (Tanemoto et al., 2000).

The activity of these channels is key for DCT function. In humans, loss of function mutations in the gene *KCNJ10* (encoding Kir4.1) are the cause of EAST/SESAME (epilepsy, ataxia, sensorineural deafness, and renal tubulopathy/seizures, sensorineural deafness, ataxia, mental retardation, and electrolyte imbalance) syndrome (Bockenhauer et al., 2009; Scholl et al., 2009), a complex disease characterized, among other manifestations, by hypokalemic metabolic alkalosis, hypomagnesemia, and hypocalciuria, a phenotype reminiscent of Gitelman syndrome. Accordingly, Kir4.1 global knockout mice have reduced levels of NCC (Zhang et al., 2014). As these mice display early lethality, mice with reduced Kir4.1 expression in the kidney (Malik et al., 2018) and kidney-specific-knockout mice (Cuevas et al., 2017) have also been generated and they also display decreased levels of expression and activity of NCC. DCT atrophy is also observed (Saritas et al., 2018).

Evidence supporting the role of the Kir4.1/5.1 heterotetramer in the establishment of membrane potential of DCT cells and its modulation by changes in [K^+^]_e_ include the following. Genetic disruption of *Kcnj10* in mice abolishes the basolateral K^+^ conductance of DCT cells and promotes depolarization (Zhang et al., 2014; Cuevas et al., 2017). NCC regulation by changes in dietary K^+^ content is completely blunted in kidney-specific-Kir4.1^–/–^ mice (Cuevas et al., 2017; Wang et al., 2018). Additionally, the activity of Kir4.1/Kir5.1 channels in the DCT has been shown to be modulated by [K^+^]_e_, as hyperpolarization and higher basolateral K^+^ conductance is observed in the DCTs of mice on low K^+^ diet. In contrast, high K^+^ intake decreases basolateral K^+^ currents and depolarizes DCT cells. These phenomena are not observed in cells from kidney-specific Kir4.1^–/–^ mice (Wang et al., 2018). All these findings together have led to the proposal of naming Kir4.1 as the ‘potassium sensor’ of the kidney.

Kir4.1 absence is not compensated by Kir5.1, as this latter subunit alone does not seem to form functional channels on the cell membrane (Pessia et al., 1996; Tanemoto et al., 2005). However, Kir5.1 does play an important role in establishing the sensitivity to [K^+^]_e_ of DCT cells. The phenotype of Kir5.1^–/–^ mice differs from that of Kir4.1^–/–^ mice. Mice lacking Kir5.1 have higher NCC activity, measured as thiazide-sensitive natriuresis (Paulais et al., 2011), and total and phosphorylated NCC levels (Wu et al., 2019). The DCT cells of these mice display higher basolateral K^+^ conductance and hyperpolarization compared to DCT cells of WT mice. Regulation of DCT basolateral K^+^ conductance and levels of pNCC and NCC in response to changes in dietary K^+^ content was impaired in Kir5.1^–/–^ mice (Wu et al., 2019).

Kir4.1/5.1 heterotetramers have different properties to Kir4.1 homotetramers (Pessia et al., 1996), such as increased intracellular pH sensitivity, as demonstrated by *in vitro* (Tanemoto et al., 2000; Tucker et al., 2000) and *ex vivo* experiments (Paulais et al., 2011). It has been suggested that decreased sensibility of Kir4.1 homotetramers to inhibition by H^+^ ions may explain the higher DCT basolateral K^+^ conductance of DCT cells on Kir5.1^–/–^ mice (Paulais et al., 2011).

### ClC-Kb and Its β-Subunit, Barttin

The ClC family comprises nine genes that encode four plasmalemmal Cl^–^ channels (ClC-1, -2, -Ka and -Kb) and five intracellular Cl^–^-H^+^ antiporters (ClC-3 to -7) (reviewed extensively by Jentsch and Pusch, 2018). ClC proteins assemble into homodimers, where each subunit mediates ion movement across the membrane.

ClC-Ka and ClC-Kb channels display different characteristics to the rest of the family, as they lack the ‘gating glutamate’ present in other family members and therefore, their voltage dependence is nearly ohmic (Waldegger and Jentsch, 2000). This allows them to mediate transmembrane movement of Cl^–^ over a wide range of membrane voltages, permitting the constant transepithelial transport of Cl^–^ (Jentsch and Pusch, 2018). Additionally, both ClC-Ka and ClC-kB require the presence of the β-subunit Barttin (Estévez et al., 2001), which acts like a chaperone that promotes localization of these channels in the plasma membrane (Waldegger et al., 2002).

In the kidney, ClC-Ka and -Kb channels play a prominent role. Both, human ClC-Ka (named ClC-K1 in mouse and rat) and ClC-Kb (ClC-K2 in mouse and rat) were initially identified and cloned from kidney (Kieferle et al., 1994), although their expression (as well as Barttin’s) is also observed in epithelial cells of the inner ear (Estévez et al., 2001). While the main site of expression of ClC-Ka in the kidney is the thin ascending limb of Henle’s loop, where it plays a role in urine concentration (Matsumura et al., 1999), ClC-Kb is primarily expressed in the basolateral membrane of the thick ascending limb of Henle’s loop (TAL), DCT, and α-intercalated cells of the ASDN, as shown by RT-PCR (Vitzthum et al., 2002), immunostaining (Hennings et al., 2017), and proteomics (Limbutara et al., 2020).

Loss of function mutations in *CLCNKB* (which encodes ClC-Kb) and *BSND* (encoding Barttin) are the cause of Bartter syndrome type III (Simon et al., 1997) and type IV (Birkenhager et al., 2001), respectively (see SLC12 section). These types of Bartter often share characteristics with Gitelman’s syndrome, such as normo- or hypocalciuria and blunted response to thiazides (Konrad et al., 2000; Seyberth and Schlingmann, 2011; Cruz and Castro, 2013). This suggests that ClC-Kb and Barttin activities are not only relevant for NKCC2-mediated salt reabsorption in the TAL, but also for NCC-mediated salt reabsorption in the DCT.

Patch-clamp assays performed in microdissected tubules from ClC-K2^–/–^ mice have shown that ClC-K2 constitutes the main basolateral Cl^–^ conductance of TAL and DCT cells. Accordingly, ClC-K2^–/–^ mice have a Bartter type III-like phenotype with severe renal salt and potassium wasting. Furosemide-sensitive, as well as thiazide-sensitive NaCl transport are completely abolished (Grill et al., 2016; Hennings et al., 2017). Decreased levels of total and phosphorylated NCC are also observed (Hennings et al., 2017). While global Barttin^–/–^ mice die a few days after birth because of severe dehydration (Rickheit et al., 2008), hypomorphic mice for Barttin (with low expression levels of a mutated Barttin) are able to thrive and recapitulate a phenotype similar to Bartter syndrome type IV (Nomura et al., 2011). Interestingly, in baseline conditions these mice have similar levels of NCC expression and phosphorylation to WT mice, despite being hypokalemic and hypovolemic, which suggests impaired physiological response of the DCT (Nomura et al., 2018).

As explained in the previous section, changes in [K^+^]_e_ regulate the membrane potential of the DCT, thanks to the basolateral expression of Kir4.1/5.1 channels. This affects the driving force for Cl^–^ movement through ClC-Kb channels, as suggested by the reduced basolateral Cl^–^ conductance observed in Kir4.1^–/–^ mice (Zhang et al., 2014). Therefore, low [K^+^]_e_ promotes hyperpolarization, which increases Cl^–^ efflux and decreases [Cl^–^]_i_, whereas, increased [K^+^]_e_ promotes depolarization, lowers Cl^–^ efflux, and increases [Cl^–^]_i_ (Terker et al., 2015b; Murthy and O’Shaughnessy, 2019). As explained previously in detail, [Cl^–^]_i_ is an important regulator of the WNK4-SPAK/OSR1 signaling pathway, which ultimately regulates NCC activity. The importance of [Cl^–^]_i_ as a second messenger that responds to changes in [K^+^]_e_ and translates them into modulation of NCC activity has been demonstrated *in* vivo, for example, by showing that hypomorphic Barttin mice do not upregulate NCC in the face of decreased K^+^ intake (Nomura et al., 2018).

### WNK4

In mice and humans, mutations in *WNK4* that cause kinase overexpression are the cause of Familial Hyperkalemic Hypertension (FHHt), a disease that is mainly the consequence of the upregulation of NCC activity (Wilson et al., 2001; Lalioti et al., 2006; Yang et al., 2007; Shibata et al., 2013). WNK4 expression has been reported in different tissues (Kahle et al., 2004; Murillo-de-Ozores et al., 2018) and in different renal cell types (Ohno et al., 2011), although in some reports definitive proof of antibody’s signal specificity by comparison with WNK4^–/–^ samples was lacking. The strictly renal origin of the FHHt phenotype suggests that absence of WNK4 activity in extrarenal tissues can be compensated probably by the activity of other WNK kinases.

Within the DCT WNK4 appears to be the major active WNK kinase. Recent evidence suggests that under basal, physiologic conditions WNK4 and KS-WNK1 (the truncated, kinase inactive version of WNK1) are probably the only WNK kinases expressed in DCT cells. Thus, WNK4 is the only WNK kinase that can phosphorylate SPAK and OSR1 in these cells. For instance, in WNK4^–/–^ mice NCC phosphorylation levels are completely ablated and a Gitelman-like syndrome is developed (Castaneda-Bueno et al., 2012). Accordingly, immunofluorescent staining using an antibody that detects phosphorylation at the S-motif serine of all WNK kinases shows that no signal is observed in kidney sections from WNK4^–/–^ mice (Thomson et al., 2020). This suggests that the catalytically active WNK in DCT is WNK4 and that no other catalytically active WNK kinase becomes activated to compensate for its absence. Additionally, in mice carrying the FHHt mutation R528H in KLHL3 that prevents WNK degradation, knocking down WNK4 completely impairs NCC phosphorylation, even when WNK1 expression levels observed in Western blot (probably KS-WNK1 in DCT and perhaps L-WNK1 in other nephron segments) remain upregulated (Susa et al., 2017).

WNK4 is also essential for low K^+^-mediated activation of NCC. No upregulation of NCC phosphorylation is observed in WNK4^–/–^ mice (Castaneda-Bueno et al., 2014; Yang et al., 2018) and, consequently, mice develop severe hypokalemia when maintained on a low K^+^ diet (Castaneda-Bueno et al., 2014).

DCT’s [Cl^–^]_i_ has been estimated to be relatively low, ranging between 10 and 20 mM (Beck et al., 1988; Boettger et al., 2002; Weinstein, 2005; Terker et al., 2015b). Works by Bazua-Valenti et al. (2015) and Terker et al. (2015a) have shown that WNK4 is more sensitive to inhibition by Cl^–^ than its related kinases WNK1 and WNK3. In *in vitro* kinase assays performed by Terker et al. (2015a) it was shown that WNK4 activity was inhibited even by the lowest [Cl^–^] tested which was 10 mM, whereas inhibition of WNK1 and WNK3 only began to be observed when [Cl^–^] reached 112 and 150 mM, respectively. These observations indicate that indeed WNK4’s Cl^–^ sensitivity lies within the range observed for [Cl^–^]_i_ in the DCT, and thus, makes it the appropriate WNK to be expressed in this cell type to allow modulation of WNK activity in response to changes in [Cl^–^]_i_.

As mentioned before, mutations in the Cl^–^ binding domain of WNK kinases (L369F/L371F and L322F/L324F mutations in human WNK1 and human WNK4, respectively) make them insensitive to Cl^–^ and constitutively active (Piala et al., 2014; Bazua-Valenti et al., 2015; Terker et al., 2015a). Introduction of these mutations in a genetic mouse model has provided the definitive proof that under basal conditions WNK4 is indeed inhibited by Cl^–^ within DCT cells, because these mice display increased NCC phosphorylation and an FHHt-like phenotype (Chen et al., 2019). Interestingly, administering a low K^+^ diet or an acute K^+^ load by oral gavage did not promote the expected increase or decrease, respectively, in NCC phosphorylation levels, supporting the idea that modulation of WNK4 kinase activity by intracellular Cl^–^ is behind the signaling mechanism implicated in such regulation.

### SPAK and OSR1

In cultured HEK293 cells, incubation with low [K^+^] media promotes an increase in pSPAK/OSR1 levels. This increase is secondary to the intracellular Cl^–^ depletion that is induced by low [K^+^]_e_ (Terker et al., 2015b). In mice, dietary K^+^ restriction induces an increase in renal SPAK/OSR1 phosphorylation levels. In DCT, apical localization of SPAK, OSR1, and pSPAK/OSR1 increases, as well as localization in cytoplasmic puncta (WNK bodies) whose formation is induced in conditions that promote pathway activation (Thomson et al., 2020). The increase in renal pSPAK/OSR1 is also observed when kidney slices are incubated on a low [K^+^] medium, suggesting that a direct effect of extracellular [K^+^] on DCT cells is implicated (Penton et al., 2016).

SPAK^–/–^ mice and SPAK knockin mice carrying a mutation that prevents phosphorylation of the T-loop’s Thr243 that is essential for kinase’s activation, both display lower levels of expression and phosphorylation of NCC and a Gitelman-like syndrome (Rafiqi et al., 2010; Yang et al., 2010; Lin et al., 2011; McCormick et al., 2011; Grimm et al., 2012). In contrast, kidney specific OSR1^–/–^ mice display normal to higher levels of pNCC (Lin et al., 2011; Ferdaus et al., 2016) and a Bartter-like phenotype with reduced pNKCC2 levels. These observations led to the idea that SPAK mainly participates in NCC regulation, while OSR1 may be more important for NKCC2 regulation. However, even though OSR1 cannot fully compensate to maintain NCC activity in the absence of active SPAK, several observations support the notion that OSR1 is also a physiological modulator of NCC.

First, Terker et al. (2014) showed that OSR1, but not SPAK is essential for β-adrenergic stimulation of NCC. Second, Chiga et al. (2011) showed that the FHHt phenotype of WNK4^D561A/+^ mice was not fully corrected by inactivation of SPAK (in WNK4^D561A/+^SPAK^T243A/T243A^ mice). However, inactivation of one copy SPAK and one copy of OSR1 (WNK4^D561A/+^ SPAK^T243A/+^ OSR1^T185A/+^ mice) did normalize blood pressure, plasma [K^+^], and pNCC levels (Chiga et al., 2011). Third, Ferdaus et al. (2016) showed that, whereas in SPAK^–/–^ mice and kidney specific OSR1^–/–^ mice an increase in pNCC was observed when placed on K^+^ deficient diet, double knockout mice were unable to upregulate NCC phosphorylation under this condition. Accordingly, plasma [K^+^] levels were significantly lower in the double mutants than in the single mutants. The severe hypokalemia developed in the double knockouts under dietary K^+^ restriction was reminiscent to the one observed in WNK4^–/–^ mice on this same condition (Castaneda-Bueno et al., 2014). These results highlight the importance of the WNK4-SPAK/OSR1 signaling pathway for NCC activation and maintenance of K^+^ homeostasis under K^+^ deprivation. Finally, supporting this view, Grimm et al. (2017) recently showed that constitutive activation of SPAK exclusively in the DCT is sufficient to develop hyperkalemia, secondary to the activation of NCC.

## Cation-Chloride Cotransporters in the Regulation of the Neuronal Response to GABA

The [Cl^–^]_i_ and its regulation by a diverse family of Cl^–^ transporters is a crucial factor affecting GABAergic transmission during brain development and in the mature nervous system. In most neurons, [Cl^–^]_i_ concentration is largely dependent on the activity of two cotransporters of the CCC family: KCC2 and NKCC1, although KCC3 is also an important cotransporter in the CNS. The activity of these transporters can decrease or increase neuronal [Cl^–^]_i_, respectively, and therefore they can alter the polarity (inhibitory or excitatory) and the magnitude of GABAergic transmission. The abnormal function of these transporters can lead to neurologic disorders, including developmental disorders, epilepsy, schizophrenia, and autism. Knowledge of the expression levels and functional regulation of these Cl^–^ transporters in the central nervous system is crucial to understand the basis for Cl^–^ homeostasis under normal and pathological conditions. For reviews see Ben-Ari et al. (2007); Blaesse et al. (2009), Kaila et al. (2014); Titz et al. (2015), Kahle and Delpire (2016), and Ben-Ari (2017).

**FIGURE 6:**
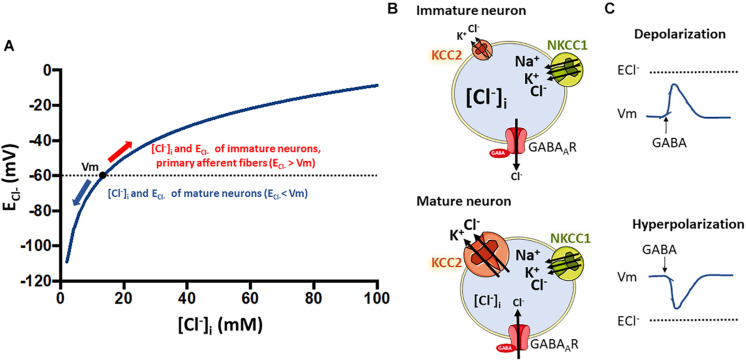
Relationship between intracellular chloride concentration ([Cl^–^]_i_) and the equilibrium potential for chloride (E_Cl–_) that determine the type of response to GABA stimulation in neurons. **(A)** Shows how the E_Cl–_ is affected by changes in [Cl^–^]_i_ according to the Nernst equation. Resting membrane potential in neurons at physiological conditions is around –60 mV. The [Cl^–^]_i_ of neurons changes along development with higher levels in immature neurons that decrease as they develop into mature cells (Ben-Ari, 2002). This decrease is coupled to the developmental upregulation of KCC2 expression (Rivera et al., 1999). The low KCC2 expression and activity in immature neurons **(B)**, and thus the high NKCC1 to KCC2 activity ratio, is responsible for the observed higher [Cl^–^]_i_ levels (around 20–40 mM) (Kakazu et al., 1999; Ben-Ari, 2002; Yamada et al., 2004). At these levels of [Cl^–^]_i_, E_Cl–_ is higher than Vm **(A)** and GABA stimulation of GABA_A_R receptors promote Cl^–^ influx and neuronal depolarization **(C)**. Conversely, in mature neurons the upregulation of KCC2 expression decreases the NKCC2 to KCC2 activity ratio **(B)**. This sets the [Cl^–^]_i_ at a lower value (around 5–12 mM) and thus, E_Cl–_ is now lower than the Vm and GABA becomes an inhibitory neurotransmitter. As an exception, primary afferent neurons conserve a high NKCC1 to KCC2 activity ratio all the way through adulthood (Alvarez-Leefmans, 2010). Thus, GABA stimulation of their terminal synapses produce a depolarizing response that is responsible for pre-synaptic inhibition, a mechanism that modulates the input of painful signals from the periphery.

The ability of neurons in the CNS to inhibit each other is just as important as the ability to excite each other. While several excitatory neurotransmitters exist (glutamate, acetylcholine, ATP etc.), neuronal inhibition is mainly mediated by γ- aminobutyric acid (GABA) and to lesser extent by glycine. GABA binds to Cl^–^-permeable GABA_A_ receptors (GABA_A_R) and their resultant activation leads to the opening of the receptor’s ion channel, resulting in Cl^–^ movement. The direction of the ion flux depends on the electrochemical driving force acting on the Cl^–^ ions, which is the difference between the cell’s membrane potential (Vm) and the Cl^–^ equilibrium potential (E_Cl–_). The latter depends on the [Cl^–^] gradient across the cell membrane. With a [Cl^–^]_i_ of 8 mM, the E_Cl–_ is about -70 mV (Figure 6). This value is often more negative than the neuron resting Vm (Kakazu et al., 1999; Rivera et al., 1999; Yamada et al., 2004; Glykys et al., 2014).

The regulation of neuronal [Cl^–^]_i_ by CCCs is essential for the normal activity of many neural circuits. In the mature brain, neuronal [Cl^–^]_i_ is low, and GABA binding to their postsynaptic receptors leads to Cl^–^ influx and post-synaptic hyperpolarization, which moves Vm away from the firing threshold, causing inhibition of excitability. Conversely, in the immature brain, [Cl^–^]_i_ is significantly higher, so E_Cl–_ is more positive than Vm, and GABA produces Cl^–^ efflux, depolarizing responses, and increased excitability by moving Vm closer to the firing threshold (Ben-Ari, 2002).

The transition of GABA_A_ responses, from excitatory in immature neurons and neurons precursors to inhibitory in mature neurons, occurs because [Cl^–^]_i_ decreases and E_Cl–_ shifts in the negative direction due to the high expression of NKCC1 (mediating Cl^–^ influx) and low expression of KCC2 (mediating Cl^–^ efflux) in immature neurons and the strong developmental upregulation of KCC2 in mature ones (Rivera et al., 1999; Ben-Ari et al., 2007). Onset of developmental upregulation of KCC2 seems to be species-specific, for example, occurring postnatally in rats (Rivera et al., 1999), and during the second half of gestation in humans (Vanhatalo et al., 2005; Sedmak et al., 2016) (for review see Kaila et al., 2014). In the mature mammalian nervous system, KCC2 is highly expressed in most central neurons, but absent or expressed at low levels in peripheral neurons and in other nervous cell-types (Payne et al., 1996; Li et al., 2002).

### NKCC1 and KCC2 Modulate Circadian Rhythms Determined by GABA

Another example of reversal of GABAergic responses, but that occur in a shorter timescale, has been reported in neurons from the suprachiasmatic nuclei (SCN). The reversal potential of GABAergic postsynaptic currents of these cells (the potential at which GABA responses changes from hyperpolarizing to depolarizing), displays diurnal variations of about 30 mV, suggesting daytime versus nighttime differences of [Cl^–^]_i_ levels (Irwin and Allen, 2009). Recent works have shown that NKCC1 expression in the SCN of the Syrian hamster is regulated by environmental light and displays circadian changes, suggesting that this may determine GABA polarity in a circadian manner (McNeill et al., 2020).

Similarly, in serotonergic neurons of the dorsal raphe nucleus (DRN), which participate in the sleep-wake cycle, GABAergic inhibition displays circadian variations. At daytime, hyperpolarizing responses to the GABA_A_R agonist muscimol are larger, and their equilibrium potential more negative compared to those measured at nighttime. Coincidently, the expression of KCC2 (mediating Cl^–^ efflux) is higher during daytime than that during nighttime, with no changes in expression pattern of NKCC1 (mediating Cl^–^ influx). Expression levels of the neuronal NO synthase (nNOS), present in most serotonergic DRN neurons, are higher at daytime than at night-time, and in brain slices treated with the NO donor sodium nitroprusside (SNP) the expression of KCC2, WNK1, WNK2, WNK3, SPAK, and OSR1 in the DRN increased, whereas phosphorylated SPAK decreased. Together, these results suggest that modulation of GABAergic inhibition of wake-inducing DRN neurons during the sleep-wake cycle is regulated by circadian variations in nNOS-derived NO concentration that in turn affect the WNK-SPAK/OSR1-KCC2 signaling (Kim et al., 2018).

### Roles of KCC2 in the CNS: Lessons Learned From Genetic Mouse Models and Human Mutations Associated With Disease

KCC2^–/–^ mice, lacking both KCC2a and KCC2b isoforms, exhibit elevated [Cl^–^]_i_ and GABA-induced neuronal excitation throughout the nervous system (Hubner et al., 2001) (Table 3). They die shortly after birth due to severe motor abnormalities that cause respiratory failure. Brainstem preparations of E18.5 KCC2^–/–^ failed to show respiratory-related motor output of the pre-Bötzinger complex, a cluster of interneurons in the medulla that participate in the generation of respiratory rhythm. Treatment of medullary slices from newborn (P0–P7) WT mice with the KCC2 inhibitor (Dihydroindenyl)oxy alkanoic acid (DIOA) has also been shown to decrease the frequency of the respiration-related rhythmic activity (Okabe et al., 2015). KCC2b^–/–^ mice (the most abundant isoform in the nervous system) exhibit frequent generalized seizures that cause their death between postnatal days 12 and 17 (Woo et al., 2002). In these mice, KCC2a expression is intact (this isoform is produced from an alternative promoter and has an alternative exon 1 that was not targeted by the knockout strategy) and is estimated to represent between 5-10% of the normal total KCC2 expression in the mature brain cortex (Uvarov et al., 2007). Thus, the residual KCC2 activity is thought to explain the slight phenotypic differences between the two knockout models (Gagnon and Delpire, 2013). KCC2b^+/–^ mice show reduced KCC2 expression and can reach adulthood but are prone to suffer epileptic seizures (Woo et al., 2002).

**TABLE 3:** Genetically engineered mouse models with mutations in KCC2.

Mouse model	Mutation	Effect on protein expression or function	Phenotype	References
KCC2 ^–/–^	Elimination of exon 5	Complete absence of KCC2 expression	Neonatal death due to inability to breath, severe motor deficits, abnormal motoneuron activity due to excitatory response to GABA.	Hubner et al., 2001
KCC2b ^–/–^	Elimination of exon 1	Absence of KCC2b, but not KCC2a expression	Die 12–17 days after birth. Abnormal posture (stiff limbs), frequent generalized seizures leading to brain injury, neuronal hyperexcitability (measured in hippocampal CA1 pyramidal neurons).	Woo et al., 2002
KCC2b^+/–^	Elimination of exon 1, heterozygous	Decreased expression of KCC2b isoform	Increased susceptibility to the proconvulsant pentylenetetrazole, sporadic seizures in aging mice.	Woo et al., 2002
KCC2^E/E^	Phosphomimetic T906E/T1007E mutations, homozygous	Decreased KCC2 activity	Neonatal death due to inability to breath. In cesarean section-delivered mice at E18.5: spontaneous and touch-evoked generalized seizures. Abnormal neuronal distribution. Lower frequency of locomotor rhythm measured in lumbar 2 ventral roots.	Watanabe et al., 2019
KCC2^A/A^	Phosphoablative T906A/T1007A mutations, homozygous	Increased KCC2 activity	Survive through adulthood with no overt phenotypes. Normal gross brain morphology and neuronal excitability. More negative E_GABA_ measured in hippocampal neurons. Delay of kainate-induced seizure onset and decrease in mortality rate from status epilepticus.	Moore et al., 2018
KCC2^S940A/S940A^	Phosphoablative S940A mutation, homozygous	No effect on basal KCC2 activity in hippocampal neurons, but decreased KCC2 activity in glutamate stimulated neurons	Reach adulthood with no overt phenotypes. Increased sensitivity to kainate: accelerated onset of status epilepticus and increased seizure severity.	Silayeva et al., 2015

Phosphorylation of KCC2 by the WNK-SPAK/OSR1 downregulates its activity, reducing the rate of Cl^–^ extrusion. Thus, if KCC2 phosphorylation is stimulated, GABAergic inhibition is expected to be weaker or null and the polarity of GABAergic responses could even reverse from inhibitory to excitatory. For instance, KCC2 phosphorylation in Thr906/Thr1007 by WNK1 decreased Cl^–^ extrusion and promoted GABAergic depolarization in cultured mature neurons (Inoue et al., 2012). Moreover, dephosphorylation of KCC2 in brain mouse has been shown to parallel the reversal of GABAergic responses (Friedel et al., 2015; Watanabe et al., 2019).

In the mouse model KCC2^E/E^ that expresses a KCC2 cotransporter harboring the phosphomimetic T906E/T1007E mutations in both alleles, touch-evoked epilepsy, disrupted locomotor rhythmicity, absence of spontaneous respiratory discharges in cervical spinal cord neurons, and early death due to respiratory arrest were reported (Watanabe et al., 2019). It has been shown that the disruption in the developmental switch in polarity of GABAergic transmission can affect normal neuronal proliferation, migration, and dendritic spine maturation (Li et al., 2007; Ben-Ari et al., 2012). Accordingly, KCC2^E/E^ mice presented anomalous neuronal distribution, but dendritic spine morphology was normal.

In contrast, in KCC2^T906A/T1007A^ mice, in which mutations mimic a permanent dephosphorylated (hyperactive) state of KCC2, a reduction in kainate-induced epileptic seizures was observed, possibly due to stronger inhibitory GABAergic synapsis throughout the CNS (Moore et al., 2018). Altogether, these results suggest that adequate KCC2-dependent Cl^–^ extrusion is essential for the correct function of a variety of neuronal circuits, and that its impairment or dysfunction causes inappropriate neuronal locomotor rhythmogenesis and touch-evoked epileptic seizures.

In humans, mutations that indirectly impair KCC2-Ser940 phosphorylation (R952H and R1049C) have been associated with idiopathic epilepsy (Kahle et al., 2014) and familial febrile seizures (Puskarjov et al., 2014). This latter condition associates with abnormal dendritic spine formation. While KCC2 phosphorylation in Thr residues (Rinehart et al., 2009; de los Heros et al., 2014) and Tyr residues (Watanabe et al., 2009; Lee et al., 2010) decrease its membrane availability and rate of ion transport, KCC2 phosphorylation in Ser940, mediated by protein kinase C (PKC), is associated with KCC2 stability in the plasma membrane and increased Cl^–^ transport (Lee et al., 2007). Thus, KCC2 phosphorylation in Ser940 leads to reduced [Cl^–^]_i_ and stronger GABAergic inhibition (Lee et al., 2007, 2011). In cultured cortical rat neurons, glutamate-mediated NMDA receptor activation triggers Ca^2+^ influx and PP1 activation that in turn mediates KCC2-Ser940 dephosphorylation (Lee et al., 2011). This leads to diminished Cl^–^ extrusion and weaker GABAergic inhibition. However, if Ser940 dephosphorylation is blocked by treatment of cultured neurons with okadaic acid, the downregulation of KCC2 is prevented and the strength of GABAergic inhibition is unaffected. When Ser940 phosphorylation is prevented in vivo in the KCC2^S940A/S940A^ mouse, no effect is observed on basal Cl^–^ extrusion in hippocampal cultured neurons, but decreased KCC2 activity is observed in glutamate stimulated neurons. Onset of kainate-induced status epilepticus is accelerated and seizure severity is increased in these mice (Silayeva et al., 2015).

In mature neurons in culture, GABA_A_R activation correlates with increased expression of KCC2 in the plasma membrane and KCC2 dephosphorylation at Thr906/Thr1007 (Heubl et al., 2017). Activation of GABA_A_R allows Cl^–^ influx that significantly increases [Cl^–^]_i_. This in turn, as a homeostatic mechanism, turns off the WNK-SPAK/OSR1 pathway leading to increased membrane KCC2 expression, thus, its activity, favoring Cl^–^ extrusion. The opposite effect occurs when an antagonist blocks GABA transmission and produces activation of the WNK-SPAK cascade. [Cl^–^]_i_ depletion induces activating phosphorylation of WNK1 at Ser382, SPAK at Ser373, and inactivating phosphorylation of KCC2 at Thr906/Thr1007. This also increases NKCC1 phosphorylation at sites Thr203, Thr207, and Thr212. All these events promote net Cl^–^ influx. Thus, modulation of the WNK-SPAK/OSR1 pathway by [Cl^–^]_i_ is an important mechanism for restoration of [Cl^–^]_i_ levels after GABA_A_R activation or blockade.

### Association of CCCs Activities in the Development of Schizophrenia and Autism

The dysregulation of NKCC1 and KCC2 activity with the consequent altered [Cl^–^]_i_ homeostasis has been related to psychiatric disorders like autism and schizophrenia in which GABA induced inhibition is altered. Rare genetic variants in *SLC12A5* (encoding KCC2) that decrease the KCC2-mediated Cl^–^ extrusion have been linked to autism (R952H and R1049C) and schizophrenia (R952H) (Merner et al., 2015) and a gain of function missense variant in *SLC12A2* (encoding NKCC1; Y199C) has been reported in a large cohort of schizophrenic patients (Merner et al., 2016). In addition, Hyde et al. (2011) showed that the NKCC1/KCC2 expression ratio in the hippocampal formation of human brains from schizophrenic patients is higher than that observed in non-schizophrenic subjects. Furthermore, post-mortem analyzed brains from schizophrenic patients show higher transcript levels of *OXSR1* (coding OSR1 kinase) and *WNK3* (Arion et al., 2011). *A* role of NKCC1 in the pathophysiology of these brain diseases is also evidenced by the beneficial effects observed with bumetanide (an NKCC1 inhibitor) administration (Lemonnier et al., 2016, 2017; Merner et al., 2016; Ben-Ari, 2017; Rahmanzadeh et al., 2017; Kharod et al., 2019; Mollajani et al., 2019; Zhang et al., 2020). In three different clinical trials, bumetanide administered to autistic patients showed significant improvement of the autistic symptoms and signs (Lemonnier et al., 2017; Zhou et al., 2020). These effects correlated with an increase in the GABA/Glutamate synapsis ratio recorded using magnetic resonance microscopy in the human insular cortex (Zhang et al., 2020). Encouraging effects of bumetanide have also been reported in schizophrenic patients (Lemonnier et al., 2016; Merner et al., 2016; Rahmanzadeh et al., 2017).

### KCC2 and NKCC1 Modulate Peripheral Sensory Transmission

**FIGURE 7:**
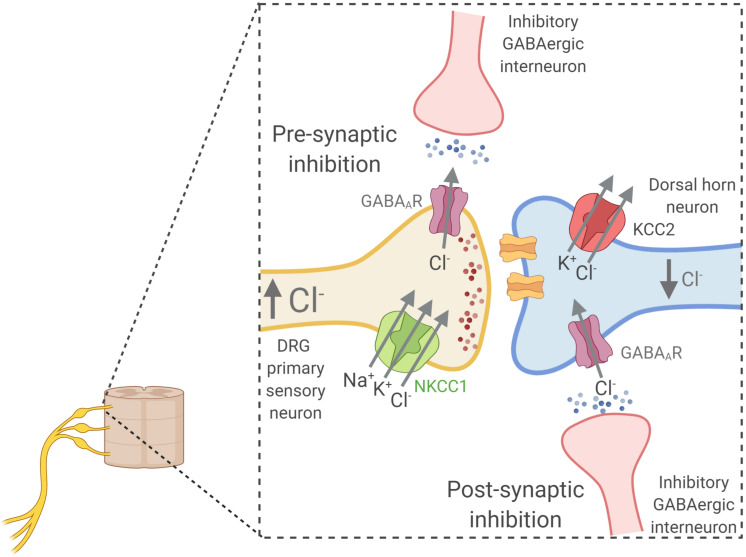
Simplified model of the role of CCCs in the modulation of pain perception. The high NKCC1 to KCC activity ratio in dorsal root ganglia (DRG) primary sensory neurons sets the [Cl^–^]_i_ at high levels (∼40 mM) (Alvarez-Leefmans, 2010). Thus, in these cells, the E_Cl–_ is less negative than the Vm and stimulation of GABA_A_R located in their synaptic terminals by GABA released from terminals of inhibitory interneurons leads to Cl^–^ efflux and primary afferent depolarization (PAD). PAD inhibits glutamate release preventing excitation of dorsal horn neurons. This mechanism is known as pre-synaptic inhibition. In contrast, dorsal horn neurons, like other CNS neurons, have a low [Cl^–^]_i_ due in large part to a high KCC2 activity. Thus, GABA released from terminals of inhibitory interneurons stimulate GABA_A_R on the post-synaptic membranes of dorsal horn neurons and this leads to GABA_A_R mediated Cl^–^ influx and hyperpolarization of the post synaptic membrane, reducing its excitability. This mechanism is known as post-synaptic inhibition. Pre- and post-synaptic inhibition are particularly important mechanisms for the modulation of spinal nociceptive processing, and thus, altered function of NKCC1 or KCC2 can lead to phenotypes of altered pain perception. Created with BioRender.com.

CCCs are also expressed in neurons of the spinal cord and peripheral nervous system, where they play an important role in the processing of somatosensory information (Kahle et al., 2008; Alvarez-Leefmans, 2010). The observed effects of altered CCC function in different models suggest a particularly important role in the inhibition of spinal nociceptive processing (Sung et al., 2000; Coull et al., 2003; Laird et al., 2004; Tornberg et al., 2005; Gagnon and Delpire, 2013). The peripheral processes of primary sensory neurons (PSN), whose soma is located in the dorsal root ganglia (DRG), collect information from nociceptive receptors throughout the body. Their central processes conduct sensory signals into the dorsal horn of the spinal cord, the first relay in the central nervous system where nociceptive information is integrated and then transmitted to the brain through nociceptive specific projection neurons. Integration of these signals in the brain is necessary for conscious pain perception. GABAergic inhibition is an important mechanism for modulation of pain input from the periphery. Both, primary sensory neurons of the DRG and dorsal horn neurons are subject to GABAergic inhibition (Figure 7). GABA released from synaptic terminals of inhibitory interneurons of the dorsal horn and inhibitory descending fibers can modulate spinal nociceptive processing via two mechanisms: presynaptic inhibition of PSN and post-synaptic inhibition of spinal cord projection neurons (Rudomin and Schmidt, 1999; Alvarez-Leefmans, 2010; Guo and Hu, 2014).

The pre-synaptic inhibition of PSN involves GABA-induced depolarization of their excitatory synaptic terminals that causes a reduction in neurotransmitter release due to still controversial mechanisms (Guo and Hu, 2014). This phenomenon has been called primary afferent depolarization. The depolarizing effect of GABA is possible because PSN, unlike mature neurons from the CNS, maintain [Cl^–^]_i_ above electrochemical equilibrium due to a high NKCC1/KCC2 activity ratio (Sung et al., 2000; Alvarez-Leefmans et al., 2001; Price et al., 2006; Rocha-González et al., 2008; Mao et al., 2012). On the other hand, in post-synaptic inhibition, GABAA receptor stimulation in spinal cord neurons induce hyperpolarization and thus reduce their excitability. Like in other mature CNS neurons, [Cl^–^]_i_ of these neurons is low due to greater KCC2 activity than NKCC1 activity (Price et al., 2005).

Dysregulation of CCCs function in PNS and spinal cord neurons that affect both inhibitory mechanisms has been associated to the pathogenesis of neuropathic pain. Regarding pre-synaptic inhibition, given that the high expression and activity of NKCC1 in DRG neurons is responsible for the high [Cl^–^]_i_ that facilitates GABA-induced depolarization (Rocha-González et al., 2008; Alvarez-Leefmans, 2010), it has been proposed that the decreased sensitivity to pain observed in NKCC1^–/–^ mice might be explained by absence of pre-synaptic inhibition (Sung et al., 2000; Laird et al., 2004; Gagnon and Delpire, 2013).

On the other hand, altered KCC2 activity in dorsal horn pain neurons has been associated to neuropathic pain related to altered post-synaptic GABAergic inhibition. Mice with reduced expression of KCC2 showed reduced sensitivity to tactile and noxious thermal stimuli (Tornberg et al., 2005). Coull et al. (2003) showed that KCC2 is highly expressed in lamina I dorsal horn pain neurons and that pharmacological blockade or knockdown of spinal KCC2 in rats reduced the nociceptive threshold. Additionally, in the model of neuropathic pain induced by peripheral nerve injury Coull et al. (2003) observed a reduction in KCC2 expression in lamina I neurons that altered the E_Cl–_ (making it less negative) and shifted the response to GABA stimulation from inhibitory to excitatory. Nomura et al. (2006) also showed a decrease in KCC2 expression in lamina I and lamina II neurons in a model of tissue injury-induced inflammatory pain. Thus, loss of post-synaptic inhibition due to KCC2 dysregulation seems to be a common mechanism underlying neuropathic and inflammatory pain. Supporting this, it has been shown that the CLP257 compound that restores Cl^–^ transport and rescues KCC2 membrane expression in the dorsal horn following nerve injury, normalized stimulus-evoked responses in spinal nociceptive pathways and alleviated hypersensitivity (Gagnon et al., 2013).

Altered regulation of KCC2 activity has also been implicated in Hereditary Sensory and Autonomic Neuropathy type 2 (HSAN2, OMIM 201300). Patients with HSAN2 suffer from severe sensory loss of heat, touch or pain perception with a partial loss of peripheral sensory nerves (Rahmani et al., 2018). This is an autosomal recessive Mendelian disease caused by mutations in the HSN2 exon of WNK1 (Shekarabi et al., 2008; Pacheco-Cuellar et al., 2011). Interestingly, the case of a female patient has been reported who is a compound heterozygote for a 1 bp deletion in the HSN2 exon and a 2 bp deletion in exon 6 of the WNK1 gene (Shekarabi et al., 2008). The *WNK1* gene encodes different WNK1 isoforms due to tissue-specific alternative splicing (Vidal-Petiot et al., 2012). Transcripts containing the HSN2 exon (located between exons 8 and 9) have been shown to be abundant in the dorsal horn, DRG, and peripheral nerves (Vidal-Petiot et al., 2012; Shekarabi et al., 2013). In a mouse model in which HSN2-containing WNK1 transcripts are absent due to the introduction of loxP recombination sites flanking this exon, the HASN2 phenotype is not fully recapitulated, but the mice show reduced pain hypersensitivity after peripheral nerve injury (PNI) (Kahle et al., 2016). PNI increased WNK1-HSN2 expression and KCC2 phosphorylation (at Thr906 and Thr1007) in spinal cord homogenates of wild-type mice, but this increase was blunted in WNK1^ΔHSN2/ΔHSN2^ mice. Accordingly, the reversal potential for GABA-induced currents (E_GABA_) measured in lamina II neurons of spinal cord slices was less negative in slices from WT mice with PNI than in those from sham operated mice. E_GABA_ was restored to more negative values by incubation of slices with the WNK-SPAK pathway inhibitor STOCK1S-50699. Moreover, E_GABA_ of lamina II neurons from WNK1^ΔHSN2/ΔHSN2^ mice was not altered after PNI. Thus, the PNI-induced increase in KCC2 phosphorylation is dependent on WNK1-HSN2 activity and the resulting decrease in KCC2 activity promotes an increase in [Cl^–^]_i_ of spinal cord neurons that shifts the E_GABA_ to more positive values.

### Association of SPAK in Body Weight Control

Finally, there is a growing interest in understanding the importance of GABAergic transmission in regulating body weight balance (Tong et al., 2008; Kim et al., 2015; Sohrabipour et al., 2018). GABAergic transmission in hypothalamic areas and in the brainstem participates in a neuroendocrine network that modulates caloric intake, energy expenditure, and thermogenesis according to the level of food intake (Pigeyre et al., 2016). Neurons in the arcuate nucleus (ARC) express leptin receptors (LepR), whose activation triggers GABAergic inhibition by ARC neurons of PVN neurons (Kong et al., 2012). This in turn relieves the tonic inhibition that PVN neurons exert on brown adipose tissue (BAT), thus increasing energy expenditure through BAT-dependent thermogenesis. The effects of leptin on energy expenditure and thermogenesis are mediated by RIP neurons of the ARC (Tong et al., 2008; Vong et al., 2011; Kim et al., 2015). In mice in which GABA production is specifically impaired in RIP neurons, decreased oxygen consumption and BAT activity was observed, and they gained more body weight and fat mass than their wild type littermates when placed on a high fat diet (Kong et al., 2012). Given that in most neurons a key determinant of [Cl^–^]_i_, and thus of GABAergic response, is the NKCC1/KCC2 activity ratio, it is likely that this mechanism plays also an important role in GABA-sensitive neurons of the PVN. Interestingly, we have recently observed that mice expressing an inactive form of SPAK that is unable to phosphorylate NKCC1 and KCC2 (SPAK^T243A/T243A^ mice), are resistant to developing obesity when placed on a high-fat diet despite similar levels of food intake (Torre-Villalvazo et al., 2018). These mice show higher energy expenditure, reflected by an increase in thermogenesis in the brown adipose tissue, a higher muscle mitochondrial activity, and a lower hepatic steatosis than their wild-type littermates. Thus, it is possible that this phenotype could be related to altered [Cl^–^]_i_ and response to GABA in PVN neurons.

## Additional Cellular Cl^–^ Sensing Mechanisms

Even though WNK kinases direct regulation by the anion Cl^–^ is clear now, it is likely that alternate mechanisms by which cells are able to respond to changes in [Cl^–^]_i_ exist, given that WNK kinases are not found in Bacteria and Archaea domains (Cao-Pham et al., 2018). While mammalian cells (Eagle, 1956) and some species of bacteria require Cl^–^ in order to grow (MacLeod and Onofrey, 1957; Roeßler and Müller, 1998), some other species of bacteria require Cl^–^ in order to adapt to different conditions in the environment, such as acidity or hyperosmolarity (Jordan and Davies, 2001; Roeßler et al., 2003). Cl^–^ also plays a role in signal transduction in bacteria, as it can modulate gene expression (Roeßler and Müller, 2002; Sewald et al., 2007) and enzymatic activity (Gut et al., 2006).

Accordingly, several studies have reported different genes whose expression is regulated in response to changes in [Cl^–^]_i_ in eukaryotes, such as *SCNN1A* (Niisato et al., 2004), *SCNN1B*, *SCNN1C* (Niisato et al., 2007), *COX2* (Cheng et al., 2000), *GLRX5* and *RPS27* (Valdivieso et al., 2016). GABA_A_R receptor subunits levels are also regulated by [Cl^–^]_i_ (Succol et al., 2012), The detailed mechanisms responsible for these different phenomena are still unknown, even though the kinase p38 might be involved in the regulation of *SCNN1B*, *SCNN1G* (Niisato et al., 2007) and *COX2* (Cheng et al., 2000). Interestingly, the transcription factor RUNX1 has been shown to directly bind Cl^–^ ions (Bäckström et al., 2002), but further work will be necessary to elucidate its role in the context of intracellular Cl^–^ handling, or if there are other transcription factors that respond directly or indirectly to changes in [Cl^–^]_i_.

Finally, it is not clear if other kinases might function as direct Cl^–^ sensors. While the phosphorylation of different MAP kinases, such as JNK, p38, and MEK6 is increased in response to decreased [Cl^–^]_i_ (Ohsawa et al., 2010; Wu et al., 2016), it is still unknown if these proteins are able to directly bind Cl^–^ anions, or whether its activation depends on upstream activators that sense [Cl^–^]_i_. In the case of the kinase SGK1, *in vitro* kinase activity assays suggest that SGK1 can be directly activated by increasing [Cl^–^]_i_, as incubating recombinant SGK1 with increasing concentrations of NaCl or KCl (but not Na-gluconate or K-gluconate) promotes its phosphorylation (Zhang et al., 2018). Further analysis will be helpful to determine if SGK1 is indeed able to directly bind Cl^–^ anions. Additionally, the opposite regulation of WNK kinases and SGK1 by Cl^–^ is puzzling, and it will be an interesting avenue for future investigations, especially regarding epithelial physiology, where both kinases play important roles in fluid secretion and intracellular signaling.

## Closing Remarks

In the present manuscript we reviewed the role of the Cl^–^ anion as a second messenger participating in the modulation of several physiological processes, specifically by regulating the activity of WNK kinases and their downstream signaling pathway, comprising the kinases SPAK and OSR1, as well as the cation-coupled Cl^–^ cotransporters (CCCs). Dynamic activation and inactivation of these kinases modulate the phosphorylation, and therefore activity of the CCCs. While initial description of the physiological roles of some of these proteins was facilitated by their association to genetic diseases, further investigations have shed additional light about their role in physiological and pathophysiological processes. For example, regulation of CCCs is relevant in maintaining normal cell volume in response to changes in extracellular osmolarity. In particular, the WNK3-SPAK complex seems to play an important role in cell volume regulation within the brain. Moreover, a complex [Cl^–^]_i_-sensitive signaling pathway involving basolateral ion channels and the kinases WNK4, SPAK, and OSR1 is responsible for NCC regulation by physiological plasma [K^+^], an essential process in the homeostatic modulation of renal K^+^ excretion. Finally, the WNK-SPAK/OSR1-CCC pathway has also been described to modulate GABAergic neuronal responses, where baseline [Cl^–^]_i_ dictates the direction of Cl^–^ currents elicited by binding of GABA to its receptor.

## Author Contributions

AM-d-O, MC-C, PH, GG, and MC-B wrote the manuscript, made the figures, edited the manuscript, revised and approved the final version. All the authors contributed to the article and approved the submitted version.

## Conflict of Interest

The authors declare that the research was conducted in the absence of any commercial or financial relationships that could be construed as a potential conflict of interest.
